# GPER Activation Inhibits Cancer Cell Mechanotransduction and Basement Membrane Invasion via RhoA

**DOI:** 10.3390/cancers12020289

**Published:** 2020-01-25

**Authors:** Alistair Rice, Ernesto Cortes, Dariusz Lachowski, Philipp Oertle, Carlos Matellan, Stephen D. Thorpe, Ritobrata Ghose, Haiyun Wang, David A. Lee, Marija Plodinec, Armando E. del Río Hernández

**Affiliations:** 1 Cellular and Molecular Biomechanics Laboratory, Department of Bioengineering, Faculty of Engineering, Imperial College London, South Kensington Campus, London SW7 2AZ, UK; a.rice15@imperial.ac.uk (A.R.); j.e.corteslopez@imperial.ac.uk (E.C.); d.lachowski15@imperial.ac.uk (D.L.); c.matellan16@imperial.ac.uk (C.M.); 2 Biozentrum and the Swiss Nanoscience Institute, University of Basel, 4056 Basel, Switzerland; philipp.oertle@unibas.ch; 3 Institute of Bioengineering, School of Engineering and Materials Science, Queen Mary University of London, London E1 4NS, UK; s.thorpe@qmul.ac.uk (S.D.T.); d.a.lee@qmul.ac.uk (D.A.L.); 4 Centre for Genomic Regulation (CRG), The Barcelona Institute of Science and Technology, 08003 Barcelona, Spain; rito.ghose@crg.eu; 5 School of Life Sciences and Technology, Tongji University, Shanghai 200092, China; wanghaiyun@tongji.edu.cn

**Keywords:** cancer biomechanics, metastasis, G protein-coupled receptors, tumour microenvironment

## Abstract

The invasive properties of cancer cells are intimately linked to their mechanical phenotype, which can be regulated by intracellular biochemical signalling. Cell contractility, induced by mechanotransduction of a stiff fibrotic matrix, and the epithelial–mesenchymal transition (EMT) promote invasion. Metastasis involves cells pushing through the basement membrane into the stroma—both of which are altered in composition with cancer progression. Agonists of the G protein-coupled oestrogen receptor (GPER), such as tamoxifen, have been largely used in the clinic, and interest in GPER, which is abundantly expressed in tissues, has greatly increased despite a lack of understanding regarding the mechanisms which promote its multiple effects. Here, we show that specific activation of GPER inhibits EMT, mechanotransduction and cell contractility in cancer cells via the GTPase Ras homolog family member A (RhoA). We further show that GPER activation inhibits invasion through an in vitro basement membrane mimic, similar in structure to the pancreatic basement membrane that we reveal as an asymmetric bilayer, which differs in composition between healthy and cancer patients.

## 1. Introduction

The G protein-coupled oestrogen receptor (GPER) is a transmembrane protein that induces signalling cascades within minutes in response to oestrogens. GPER activation can elicit fast downstream effects, in contrast to the slower genomic mechanisms of the nuclear oestrogen receptors (ERs). Oestrogenic signalling is a therapeutic target in the clinic, with selective oestrogen receptor modulators like tamoxifen used in the treatment of breast cancer. Tamoxifen has been identified as a specific agonist of GPER [1], and regulates morphology and contractility in fibroblasts [2], suggesting the applicability of mechanical modulation of cancer cells through GPER activation.

Cancer cell aggressiveness is particularly dependent on mechanosignalling [3] and actomyosin contractility [4], and these may be sensitive to oestrogenic signalling. Metastasis requires cells to be highly contractile following the epithelial–mesenchymal transition (EMT) [5], and external stiffness can induce EMT in cancer cells [6,7]. The invasive potential of cancer cells is linked to their rheological properties [8,9,10,11,12,13], where mechanical stress in the cytoplasm is associated with cell stiffness [14]. Oestrogenic signalling can alter the cytoskeleton of cells, with 17β-estradiol [15] and tamoxifen [16] inducing cytoskeletal remodelling and affecting cell-matrix interactions in the breast cancer cell line MCF-7. If a common mechanism could be found by which GPER activation mechanically affects the behaviour and malignancy of cancer cells, this could benefit future therapies.

Ras homolog family member A (RhoA), a GTPase which promotes contractility of the actin cytoskeleton [17], is regulated by GPER, and two mechanisms have been previously reported for this regulation. GPER activates the G protein Gαs, which can signal through either Epac or protein kinase A—both of which lead to inhibition of RhoA activity. In particular, protein kinase A phosphorylates RhoA in position serine 188 [18]. It is well-documented that phosphorylation of the serine residue 188 in the C-terminal tail of RhoA prevents its dissociation from the complex with guanine nucleotide dissociation factors (GDIs), and therefore inhibits RhoA activation [19,20]. 

The basement membrane coats the basal side of epithelial cells, and is the first physical barrier for epithelial cells in tumour dissemination [21], requiring cells to generate force to breach it [22,23]. The basement membrane is composed primarily of collagen IV and laminins [21], and has been shown in some tissues to be arranged as an asymmetric bilayer [24]. Changes in basement membrane composition are known to occur in, and promote, tumour development [25], e.g., specific laminin α-chains are associated with promoting invasion for multiple cancers [26,27].

Here, we use two different cancer cell lines (pancreatic and prostate) to delineate the mechanism through which GPER activation modulates cancer cell mechanics and invasion. Furthermore, we report the composition of the pancreatic basement membrane in healthy and pancreatic ductal adenocarcinoma (PDAC) patients, revealing a laminin/collagen IV asymmetric bilayer and disease-specific differences in laminin expression. We determine that decreased contractility in cancer cells due to GPER activation is associated with reduced invasion through a basement membrane mimic that recapitulates the in vivo basement membrane. Collectively, we demonstrate the importance of GPER in modulating the mechanical processes understood to allow invasion in metastasis.

## 2. Results

### 2.1. Reduced GPER Expression Is Associated with Cancer, Worse Survival, and Shorter Relapse-Free Time

**Figure 1:**
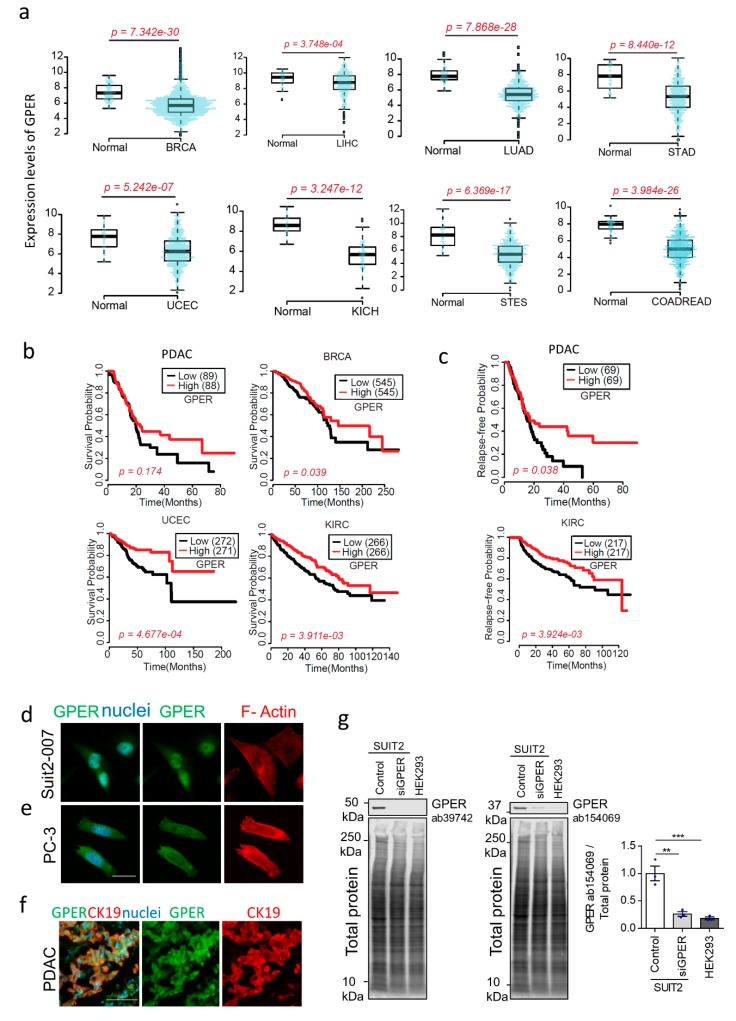
G protein-coupled oestrogen receptor (GPER) expression and correlation with survival in cancer (**a**) RNA-seq data from cancer types downloaded from TCGA cBioPortal (http://www.cbioportal.org/index.do), 11/2017 and 2/2018. Gene abundance quantified as RNA-seq by Expectation Maximisation). *p* values from Wilcoxon rank-sum test. BRCA—breast invasive carcinoma, LIHC—liver hepatocellular carcinoma, LUAD—lung adenocarcinoma, STAD—stomach adenocarcinoma, UCEC—uterine corpus endometrial carcinoma, KICH—kidney chromophobe, STES—stomach and esophageal carcinoma, and COADREAD—colorectal adenocarcinoma. Number of patients/normal—BRCA (1093/112), LIHC (371/50), LUAD (515/59), STAD (415/35), UCEC (545/35), KICH (66/25), STES (599/46), COADREAD (623/51). (**b**) Survival curves for cancer patients, divided into high and low expression determined using median gene expression of GPER. *p* values from Kaplan–Meier statistical test. PDAC—pancreatic ductal adenocarcinoma, and KIRC—Kidney renal clear cell carcinoma. For PDAC, BRCA, UCEC and KIRC, n = 177, 1090, 543, 532 patients respectively. (**c**) Relapse-free probability curves for PDAC and KIRC cancer patients. High and low expression determined using median gene expression of GPER. *p* value from Kaplan–Meier statistical test, For PDAC, KIRC n = 138, 434 patients. (**d**) Immunofluorescence images of GPER (green), actin (red), and DAPI (blue) in Suit2-007 cells. Scale bar = 25 µm. (**e**) Immunofluorescence images of GPER (green), actin (red), and DAPI (blue) in PC-3 cells. Scale bar = 25 µm. (**f**) Immunofluorescence images of GPER (green), cytokeratin 19 (red) and DAPI (blue) in PDAC patients. Scale bar = 100 µm. (**g**) Western blot for GPER and total protein in untreated SUIT2 cells (Control), SUIT2 cells with siRNA to GPER (siGPER) and HEK293 cells. Quantification of GPER (ab154069) normalised to total protein. Mean ± s.e.m. with individual values overlaid (n = 3); one-way ANOVA with Dunnett pairwise comparisons. ** *p* < 0.01, *** *p* < 0.001. Full blot images in Supplementary Figure S1.

To demonstrate the clinical relevance of GPER in cancer, we compared the level of GPER expression in human tissues from healthy and cancer patients. We downloaded and analysed RNA-seq and clinical data from the TCGA cBioPortal, quantified gene abundance by RSEM (RNA-seq by Expectation Maximisation) [28], and compared healthy and cancer samples by the Wilcoxon rank-sum statistical test. We observed that GPER expression was significantly decreased in multiple cancer types (breast, liver, lung, stomach, uterine, kidney, and colorectal) with respect to their healthy counterparts (Figure 1a).

We also plotted survival curves for multiple cancer types, comparing the difference between patients with either high or low GPER expression, as determined by the median expression level of GPER. We found that high GPER expression was associated with significantly improved survival probability (*p* < 0.05) (Figure 1b). For pancreatic ductal adenocarcinoma, survival probability for patients who survived longer than 20 months was significantly improved with higher GPER expression (*p* = 0.015). Additionally, the relapse-free probability of kidney renal clear cell carcinoma and pancreatic ductal adenocarcinoma was significantly higher for those patients with high GPER expression (Figure 1c).

### 2.2. GPER Activation Inhibits Cell Survival and Proliferation In Vitro

Given that GPER was differentially expressed in these cancers and the implications of GPER expression levels in survival and relapse-free times, we studied the effect of GPER activation on cell survival and proliferation. First, we verified that GPER is expressed in Suit2-007 and PC-3 cells (Figure 1d–e), highly mesenchymal pancreatic and prostate cancer cell lines respectively [29]. Then, we analysed human tissue samples from PDAC patients using immunofluorescence and confirmed the expression of GPER (Figure 1f). Immunoblotting analysis revealed similar results, with high expression of GPER in control Suit2-007 cells compared to GPER knockdown (siGPER) and GPER-deficient (HEK239) cells (Figure 1g and Supplementary Figure S1). Specific activation of GPER has been observed to elicit different cell survival responses depending on cell type [1], often using the specific GPER agonist G1 [30]. G1 has been previously shown to inhibit the growth of PC-3 cells [31]. We analysed the effect of the GPER agonist G1 (1 µM) and the GPER antagonist G15 (2 µM) on cell proliferation (Ki67 expression) and viability (cell number) for both cell types. No significant decrease in proliferation (Ki67 positive nuclei) or cell viability (cell number) was observed during the first 24 h, while we observed an effect on proliferation and viability after 72 h (Supplementary Figure S2). Based on these results, 24 h was chosen as a G1 treatment time point for both cell types.

### 2.3. GPER Activation Inhibits Mechanosensing and YAP Activation In Vitro

First, we sought to characterise the effects of GPER activation on cancer cell mechanics. Mechanosensing entails a cellular response to external forces, which can include stromal rigidity and shear stress [32]. These responses require mechanosensitive receptors such as integrins to produce intracellular signals that transduce external force [3]. Forces within the ECM, which lead to mechanosignalling by cancer cells, are known to facilitate invasion [33]. 

Restructuring of the actin cytoskeleton is an essential process in mechanosensing [34] and RhoA is vital in determining the organisation and dynamics of the cytoskeleton [17]. The stiffening response of endothelial cells in response to force applied by magnetic tweezers requires active RhoA [35] and cytoskeletal remodelling has been associated with GPER in human dermal fibroblasts [36], suggesting that GPER may modulate mechanosensing through RhoA in cancer cells. We tested the cyclic adenosine monophosphate (cAMP) level (involved in Gs pathway) after GPER activation using the selective agonist G1 [37]. cAMP levels were increased by 40% compared to control samples (Supplementary Figure S4). 

**Figure 2:**
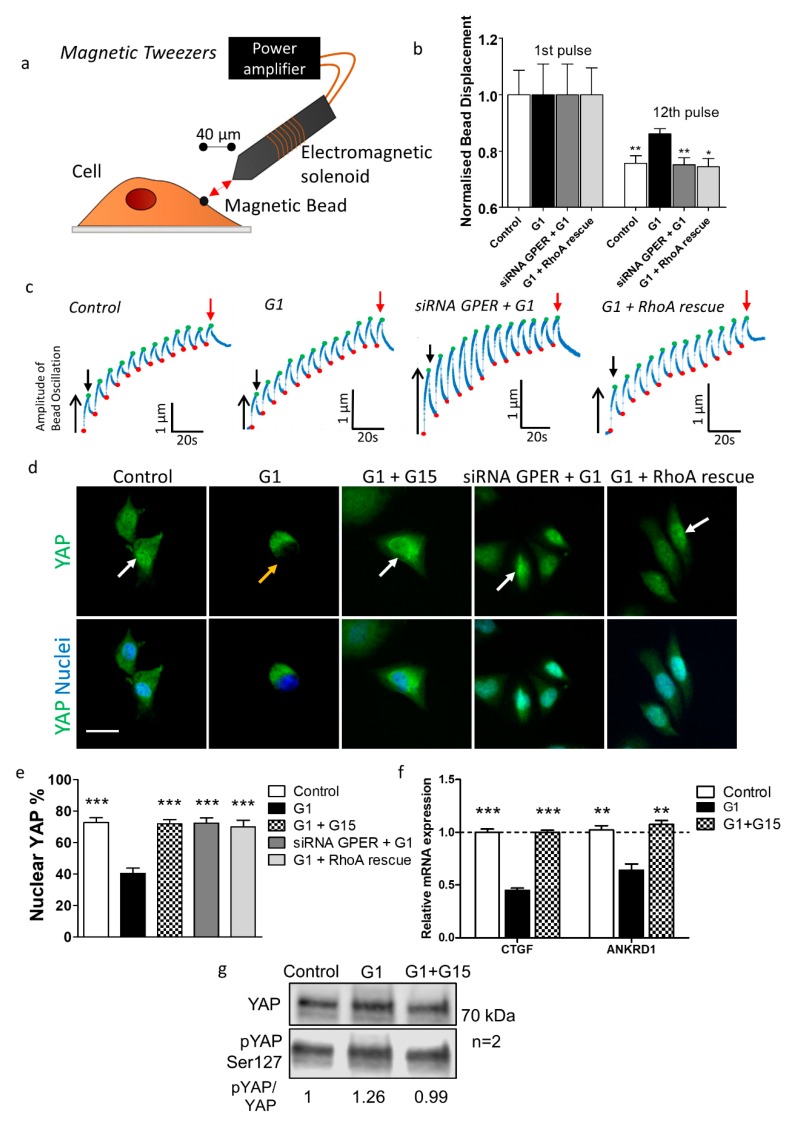
Mechanosensing in Suit2-007 cells is inhibited by GPER activation. (**a**) Diagram of magnetic tweezers setup with fibronectin-coated magnetic beads attached to cells, and tweezers placed at 40 µm lateral distance from bead. (**b**) Relative bead displacement for 1st and 12th pulses for bead profiles where displacement values for 12th pulse < 1st pulse, indicating mechanotransduction. For 1st pulse, error bars = s.e.m. for normalised displacement of bead during 1st pulse of pulsatile regime. For 12th pulse, error bars = s.e.m. for normalised bead displacement values relative to their respective 1st pulse (i.e., values between 0 and 1). For both 1st and 12th pulse, for control, G1, siRNA GPER + G1, and G1 + RhoA (Ras homolog family member A) rescue, n = 25, 16, 28 and 30 cells respectively. Mann–Whitney test comparing each 12th pulse mean to G1 12th pulse mean, * *p* < 0.05, ** *p* < 0.01. (**c**) Representative traces for bead displacement and reinforcement for control, G1, siRNA GPER + G1, and G1 + RhoA rescue conditions. Green points represent the maximum displacement in a pulse and red points the starting displacement in a pulse, which increase due to bead drift. Black downwards arrow indicates 1st pulse and red arrow indicates 12th pulse. (**d**) Immunofluorescence images of YAP (yes-associated protein 1) nuclear localisation in Suit2-007 under control, G1 (1 µM, 24 h), and G1 + G15 (1 µM and 2 µM respectively, 24 h) conditions. Green represents YAP, and blue represents DAPI staining of the nucleus. White arrow indicates nuclear localisation of YAP, orange arrow indicates cytoplasmic localisation of YAP. Scale bar = 25 µm. (**e**) Quantification of percentage cells containing nuclear YAP. For control, G1, G1 + G15, siRNA GPER + G1 and G1 + RhoA rescue, n = 24, 24, 20, 12, 12 regions of interest from three independent samples respectively. *** represents Mann–Whitney test between each individual condition and G1 condition, *p* < 0.001. (**f**) Quantitative PCR analysis of the YAP-target genes *CTGF* and *ANKRD1*, normalised to expression of *RPLP0*. Dotted line represents y = 1.0. For Mann–Whitney test between each condition and G1 condition, ** *p* < 0.01, *** *p* < 0.001. n = three independent samples. (**g**) Western Blot analysis of YAP and pYAP (phosphorylated on Ser127) for control, G1 and G1 + G15 conditions. n = two independent samples. All experiments with siRNA and RhoA were conducted 72 h after transfection. For experiments with G1, cells were treated with 1 µM G1 for 24 h before analysis. For experiments with G15, cells were treated with 2 µM G15 for 24 h before analysis.

We then assessed the ability of cancer cells to respond to external force using magnetic tweezers (Figure 2a). Control cells showed a decrease in bead displacement from 1st pulse to 12th pulse of 25%, indicating robust mechanosensing. G1-treated cells showed less reduction (14%) in bead displacement over 12 pulses, and the relative size of the 12th pulse was significantly higher than in control conditions, indicating reduced mechanosensing. When G1 was used in combination with siRNA against GPER (Supplementary Figure S3), mechanosensing recovered to control levels, indicating that the mechanical influence of G1 proceeds through GPER (Figure 2b,c, Supplementary Figure S7e,f). PC-3 mechanosensing was also inhibited by G1 treatment (Supplementary Figure S5a,b). 

Our previous results showed that GPER activation leads to a decrease in the levels of active RhoA [38,39]. In order to learn more about the mechanism by which GPER signalling inhibits RhoA activation, we focused on the two previously described pathways (via Epac or protein kinase A) [18]. We used constitutively active RhoA (Q63L) with the serine in position 188 replaced by alanine (Q63L/S188A), meaning RhoA cannot be phosphorylated in position 188. When cells rescued with this RhoA mutant were treated with G1, mechanosensing was recovered to levels comparable to control (Figure 2b,c). This indicates that serine 188 is required for the downregulation of RhoA activation via GPER and points out to a mechanism involving protein kinase A. 

YAP (yes-associated protein 1) is a mechanoresponsive transcriptional coactivator which, when activated through dephosphorylation, localises to the nucleus to regulate gene expression [40,41]. We therefore tested the effect of G1 on YAP nuclear localisation to analyse the mechanisms of mechanotransduction affected by GPER activation. G1 treatment lead to decreased nuclear localisation of YAP. The conditions G1 + the GPER antagonist G15, siRNA GPER + G1, and G1 + RhoA rescue all showed levels of YAP localisation similar to the control condition (Figure 2d,e, Supplementary Figure S5c,d, Supplementary Figure S7a,b). Accordingly, the expression of the YAP-dependent genes *CTGF* and *ANKRD1* was decreased by G1, but not with G1 + G15 (Figure 2f, Supplementary Figure S5e). Additionally, the ratio between phosphorylated YAP and non-phosphorylated YAP was increased in G1-treated Suit2-007 cells, as determined by Western Blot analysis (Figure 2g). 

Our observation that GPER activation inhibits activation of YAP suggests that other mechanical properties of the cell may be modulated by GPER activation. For this reason, we decided to study the effect of GPER activation on myosin light chain-2 (MLC-2) phosphorylation, traction force generation and cells stiffness—mechanical properties closely related to cancer cell malignancy.

### 2.4. GPER Activation Decreases Myosin-Mediated Contractility and Force Generation In Vitro

Tension within the cytoskeleton is essential for mechanosensing of external rigidity, with increased actomyosin contractility associated with cancer cell malignancy [42]. In addition to mechanotransduction, cell invasion requires myosin-induced cell contractility. MLC-2 phosphorylation, which promotes actomyosin contractility, lies downstream of RhoA [43]. We have previously reported that GPER activation reduces cell contractility and traction forces in pancreatic stellate cells [39]. Since GPER has also been observed as influential in a contractility response in human myometrium [44], we have investigated whether GPER activation can affect cell contractility in cancer cells.

**Figure 3:**
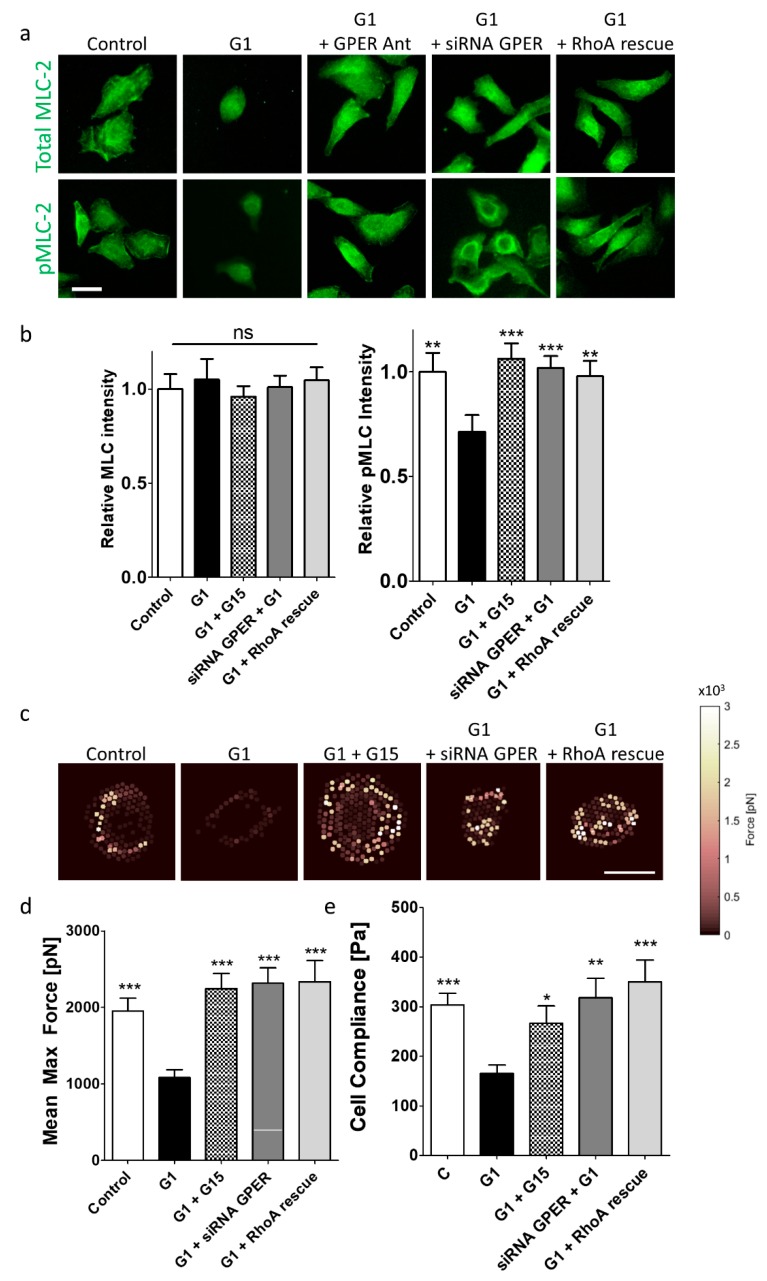
Force generation in Suit2-007 cells is inhibited by GPER activation. (**a**) Immunofluorescence staining for total MLC-2 (myosin light chain-2) and pMLC-2 (phospho-myosin light chain-2) for Suit2-007 cells. Scale bar = 25 µm. (**b**) Quantification of MLC-2 and pMLC-2 staining intensities relative to control condition. For MLC-2 intensity, control, G1 (1 µM, 24 h), G1 + G15 (1 µM and 2 µM respectively, 24 h), siRNA GPER + G1 (1 µM, 24 h), G1 (1 µM, 24 h) + RhoA rescue, n = 35, 29, 36, 36, 36 cells across three independent experiments respectively. Kruskal-Wallis analysis indicates no significant differences between median values. For pMLC-2 intensity, control, G1, G1 + G15, siRNA GPER + G1. G1 + RhoA rescue, n = 30, 24, 20, 36, 36 cells across three independent experiments respectively. Mann–Whitney test comparing each individual condition to G1-treated conditions, ** *p* < 0.01, *** *p* < 0.001. (**c**) Heat maps indicating force generation of cells on elastic pillars. Each point represents one pillar, with intensity equal to the maximum force applied to that pillar over 1 min of imaging. Scale bar = 20 µm. (**d**) Quantification of maximum mean force exerted by Suit2-007. For control, G1, G1 + G15, siRNA GPER + G1, G1 + RhoA rescue, n = 46, 44, 25, 54, 43 cells across two independent experiments respectively. Mann–Whitney test comparing each individual condition to G1-treated conditions, *** *p* < 0.001. (**e**) Young’s modulus values for control, G1 and G1 + G15 treated cells. For control, G1 and G1 + G15, n = 51, 50, and 48 cells across three independent samples respectively. * represents Mann–Whitney test, *p* < 0.05, *** *p* < 0.001. All experiments with siRNA and RhoA were conducted 72 h after transfection. For experiments with G1, cells were treated with 1 µM G1 for 24 h before analysis. For experiments with G15, cells were treated with 2 µM G15 for 24 h before analysis.

We stained Suit2-007 cells for both total MLC-2 and phosphorylated MLC-2 (pMLC-2). While total levels of MLC-2 were unchanged when comparing all conditions, pMLC-2 levels were significantly decreased in the G1 condition (Figure 3a,b, Supplementary Figure S7c,d) compared to control, G1 + G15, siRNA GPER + G1, and G1 + RhoA rescue. PC-3 cells treated with G1 showed the same trend (Supplementary Figure S6a,b). The lack of difference in MLC-2 intensities indicates that the increase in pMLC-2 intensities only comes from phosphorylation events. 

To assess the ability of cancer cells to generate force, we seeded cells on elastic pillars, which deflect in response to traction forces. Control cells exhibited a mean maximum force of approximately 2 nN. G1 treatment significantly reduced this to around 1 nN whereas G1 + G15 rescued force generation back to around 2 nN. Both siRNA GPER + G1 and G1 + RhoA rescue lead to restoration of force generation to around 2 nN (Figure 3c,d, Supplementary Figure S7g). PC-3 cells also showed a decrease in force generation following G1 treatment (Supplementary Figure S6c,d). These results indicate that GPER activation inhibits actomyosin contractility through RhoA. 

The deformability of a cell is related to its contractility, and in some cell types, invasiveness is associated with compliance, as softer cells are more able to squeeze through a membrane. To investigate the effect of GPER activation on cell deformability, cell compliance of cancer cells was measured using AFM (Atomic Force Microscopy). The Young’s modulus for Suit2-007 cells under control conditions was found to be 298 Pa, similar to the epithelial pancreatic cell line Panc-1 [45]. G1 treatment softened cells to a rigidity of 165 Pa. whereas G1+ G15 showed a rigidity of 266 Pa, a value not significantly different from the control condition. Both the conditions of siRNA GPER + G1 and G1 + RhoA rescue showed Young’s modulus values similar to control (Figure 3e). PC-3 cells also showed the same trend (Supplementary Figure S6e), with absolute Young’s modulus values within a range previously measured [46].

### 2.5. GPER Activation Inhibits the Epithelial–Mesenchymal Transition

EMT is a multifaceted phenotypic conversion associated with cancer invasiveness and progression [47], as well as cell mechanics [48]. High expression of the intermediate filament protein vimentin and nuclear localisation of the transcriptional regulator β-catenin are indicative of the mesenchymal phenotype [49]. 

**Figure 4:**
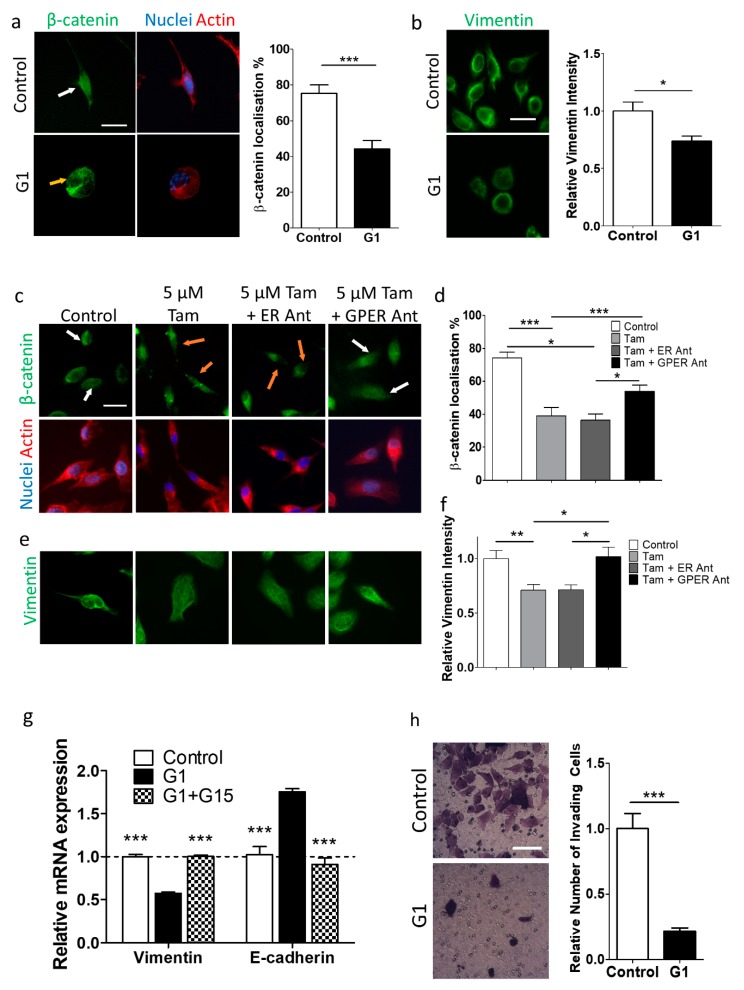
GPER activation inhibits epithelial–mesenchymal transition in Suit2-007 cells. (**a**) Left: Immunofluorescence images of Suit2-007 cells stained for β-catenin, DAPI and actin, for control and 1 μM G1 (GPER agonist) after 24 h of culture. White arrow indicates nuclear localisation of β-catenin, orange arrow indicates cytoplasmic localisation of β-catenin. Scale bar = 25 μm. Right: Quantification of β-catenin localisation. For Suit2-007, control and G1, n = 18 and 18 regions of interest respectively. For PC-3, control and G1, n = 17 and 16 regions of interest respectively. Mann–Whitney test for statistical significance, * *p* < 0.05, *** *p* < 0.001. (**b**) Left: Immunofluorescence images of Suit2-007 cells stained for vimentin, for control and G1 (1 µM) after 24 h of culture. Scale bar = 25 μm. Right: Quantification of vimentin immunofluorescence images respectively. For control and G1, n = 30 and 30 regions of interest respectively. Mann–Whitney test for statistical significance, * *p* < 0.05, *** *p* < 0.001. (**c**) Immunofluorescence images of Suit2-007 cells stained for β-catenin, DAPI and actin, for control, 5 µM tamoxifen, 5 µM tamoxifen + 1 µM ICI 182780 (ER antagonist), 5 µM tamoxifen + 2 µM G15 (GPER antagonist) for 72 h of culture. White arrows indicate nuclear localisation of β-catenin, orange arrows indicate cytoplasmic localisation of β-catenin. Scale bar = 25 μm. (**d**) Quantification of β-catenin localisation. For Suit2-007, control, 5 µM tamoxifen, 5 µM tamoxifen + 1 µM ER Ant, 5 µM tamoxifen + 2 µM GPER Ant (all for 72 h), n = 15, 11, 15, 18 regions of interest respectively. For PC-3, control, 5 µM tamoxifen, 5 µM tamoxifen + 1 µM ER Ant, 5 µM tamoxifen + GPER Ant (all for 72 h), n = 16, 15, 10, 11 regions of interest respectively. Mann–Whitney test for statistical significance, * *p* < 0.05, ** *p* < 0.01, *** *p* < 0.001. (**e**) Immunofluorescence images of Suit2-007 cells stained for vimentin, for control, 5 µM tamoxifen, 5 µM tamoxifen + 1 µM ER Ant, 5 µM tamoxifen + 2 µM GPER Ant for 72 h of culture. (**f**) Quantification of immunofluorescence intensity for vimentin. For Suit2-007, control, 5 µM tamoxifen, 5 µM tamoxifen + 1 µM ER Ant, 5 µM tamoxifen + GPER Ant (all for 72 h), n = 16, 10, 10, 17 regions of interest respectively. For PC-3, control, 5 µM tamoxifen, 5 µM tamoxifen + 1 µM ER Ant, 5 µM tamoxifen + 2 µM GPER Ant (all for 72 h), n = 17, 15, 16, 15 cells regions of interest respectively. Mann–Whitney test for statistical significance, * *p* < 0.05, ** *p* < 0.01, *** *p* < 0.001. (**g**) Quantitative PCR analysis of the YAP-target genes for vimentin and E-cadherin, normalised to expression of RPLP0. For Mann–Whitney test between each condition and G1 condition, ** *p* < 0.01, *** *p* < 0.001. n = three independent samples. **(h)** Left: Crystal Violet stained cells on Transwells for control and G1-treated conditions. Right: Count of invaded cells in imaged region for control and G1 (1 µM, 24 h) treated cells. For control and G1, n = 23 and 21 regions respectively. *** represents Mann–Whitney test, *p* < 0.001. Scale bar = 100 µm.

We observed that G1 treatment reduced β-catenin nuclear localisation and vimentin expression after 24 h (Figure 4a,b, Supplementary Figure S8a,b), indicating the loss of the mesenchymal phenotype. For the conditions G1 + G15, siRNA GPER + G1, vimentin levels were restored to levels similar to the control condition (Supplementary Figure S9). Additionally, both PCR analysis of Suit2-007 and PC-3 cells treated with G1 revealed decreased expression of vimentin and increased expression of E-cadherin at both the mRNA and protein level. Control levels of these proteins were restored with both G1 and G15 (Figure 4g, Supplementary Figure S8c).

Tamoxifen, a drug with known pharmacodynamics and a record of clinical safety [50,51], is a GPER agonist, but can also modulate the activity of the nuclear ERs [1]. Tamoxifen was observed to reduce proliferation and survival of both Suit2-007 and PC-3 cells at a concentration > 5 µM (Supplementary Figure S10). A tamoxifen concentration of 5 µM was therefore chosen as appropriate for further studies to prevent effects on proliferation.

The expression of vimentin and nuclear localisation of β-catenin were reduced following 5 µM tamoxifen treatment in both Suit2-007 and PC-3 cells. Co-incubation of tamoxifen with ICI 182780, a nuclear ER antagonist, maintained this more epithelial phenotype, whereas co-incubation with G15 showed a reversion to the mesenchymal phenotype, indicating that tamoxifen promotes the epithelial phenotype through GPER, and not the nuclear ER receptors (Figure 4c–f, Supplementary Figure S11).

### 2.6. GPER Activation Inhibits Transwell Invasion by Suit2-007 Cells

The invasive properties of cells are known to be linked to EMT [52]. Furthermore, invasion has been shown in some cells to require high cell contractility [12,13], and in other cells to require low cell stiffness [8,9,10,11]. Since G1 reduced cell contractility, cell stiffness, and EMT, we investigated its effect on invasion through collagen-coated Transwells over 24 h. Control cells showed more invasion than G1-treated cells (Figure 4h), indicating that the reduction in cell contractility by G1 may underlie the reduction in invasion.

### 2.7. Pancreas Basement Membranes Are Asymmetric Bilayers

Since G1 could inhibit invasion through collagen-coated Transwells, we considered an invasion assay that could faithfully recapitulate the in vivo basement membrane to study the effect of GPER on invasion. The basement membrane surrounding epithelial cells is the first barrier that cells must cross in metastasis [21], and is made up of various laminins and collagen IV, which provide both structural integrity and induce signalling in epithelial cells. Since cancer development is often accompanied by change in basement membrane composition and basement membrane organisation differs between organs [53], it was necessary to study the basement membrane in human pancreas, since no studies have previously attempted this characterisation. We analysed the composition of the basement membrane in pancreatic human tissues from both healthy and PDAC patients using immunofluorescence.

**Figure 5:**
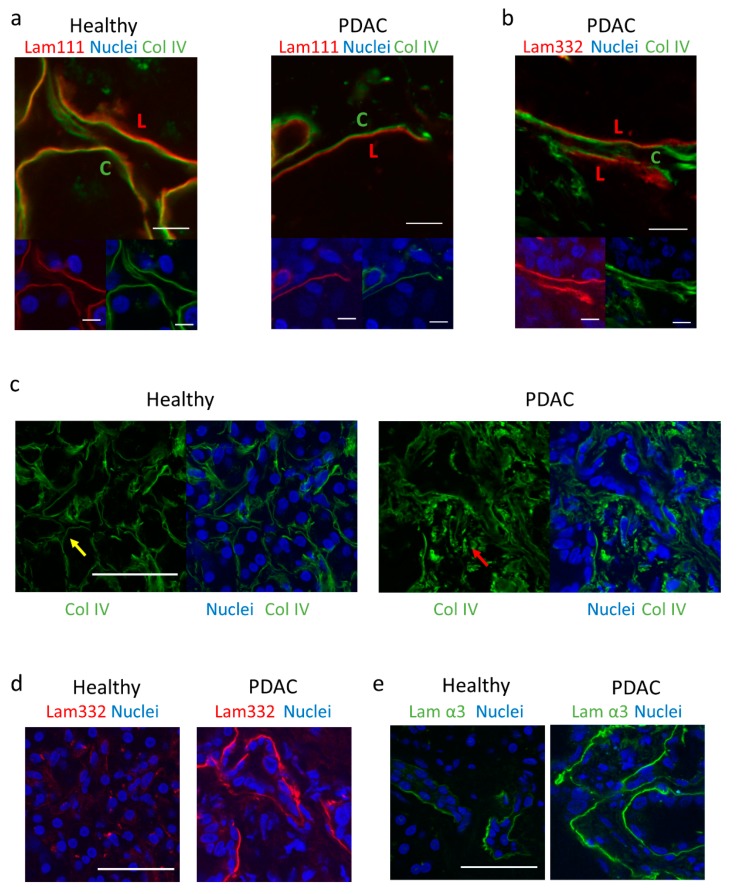
Composition of basement membrane in healthy and PDAC patients. (**a**) Immunofluorescence images of laminin/collagen IV bilayer in healthy and PDAC patients. Scale bar = 10 µm. L = laminin 111-rich side. C = collagen IV-rich side. Upper panel = Lam111 + Col IV, lower left = Lam111 + DAPI, lower right = Col IV + DAPI. (**b**) Immunofluorescence images of laminin 332/collagen IV bilayer in PDAC. Scale bar = 10 µm. L = laminin 111-rich side. C = collagen IV-rich side. Upper panel = Lam332 + Col IV, lower left = Lam332 + DAPI, lower right = Col IV + DAPI. (**c**) Immunofluorescence images of collagen IV organisation in healthy and PDAC tissues. Scale bar = 50 µm. Yellow arrow indicates organised collagen IV, red arrow indicates disorganised collagen IV. (**d**) Immunofluorescence images of laminin 332 organisation in healthy and PDAC tissues. Scale bar = 50 µm. (**e**) Immunofluorescence images of laminin α3 organisation in healthy and PDAC tissues, using P3H9 monoclonal antibody. Scale bar = 50 µm.

Immunostaining of ocular human basement membranes with laminin 111 and collagen IV has previously revealed an asymmetrical bilayer whereby laminin is located on the epithelial side and collagen IV on the stromal side, with asymmetry suggested to facilitate tissue architecture. This study further showed that multiple cell lines adhered preferentially to the epithelial side [24]. We observed that both healthy and PDAC epithelial basement membranes in the pancreas show an asymmetric bilayer with laminin 111 more highly expressed on one side, and collagen IV more highly expressed on the other (Figure 5a). Additionally, laminin 332 and collagen IV form a bilayer in PDAC tissues, indicating the consistency of the laminin-collagen asymmetry (Figure 5b).

### 2.8. PDAC Epithelial Basement Membranes Show a Disorganised Collagen IV Arrangement and Altered Laminin Expression

The fibrotic reaction is characterised by changes in the composition and organisation of the stroma and basement membrane [22]. We assessed the differences in collagen IV and laminin 111 between healthy and PDAC tissues. Collagen IV was observed to be highly organised in healthy tissues, forming an ordered network around individual acini. In contrast, PDAC tissues showed a highly disorganised collagen IV arrangement within the tissue, indicating a reduction in basement membrane integrity (Figure 5c).

We observed that healthy pancreas samples showed minimal staining of laminin 332, compared to a high intensity in PDAC samples (Figure 5d). Specific staining of the α3 chain also indicated low expression of this chain in healthy tissues, whereas higher expression was observed in PDAC samples (Figure 5e). The increased laminin presence seen here could have a role in promoting PDAC development, based on previous studies that link laminin signalling to malignancy [21,25,26,27].

### 2.9. GPER Activation Inhibits Invasion through the Basement Membrane

Invasion through the basement membrane into the surrounding stroma is the first step in metastasis. Pancreatic cancer cells have had high cell contractility linked to their ability to invade [12].

**Figure 6:**
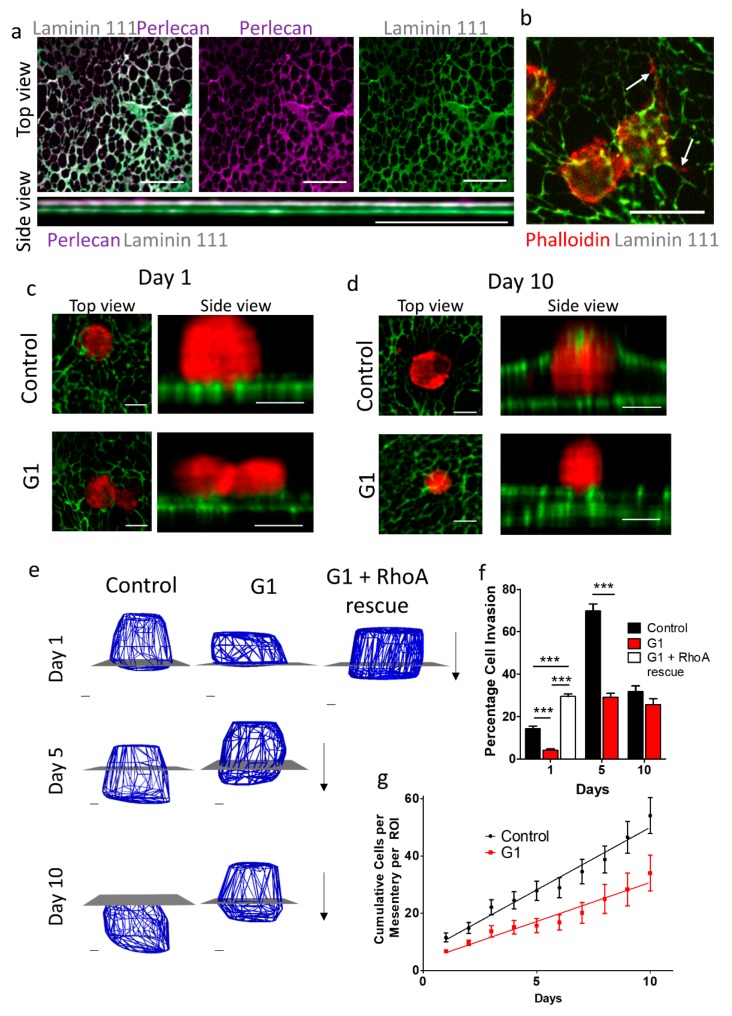
GPER activation inhibits Suit2-007 invasion through basement membrane mimics. (**a**) Immunofluorescence images of a decellularised mesentery extracted from mice, stained for laminin 111 (green) and perlecan (magenta). Image from single plane (top down view) and maximum intensity projection from side view, indicating presence of bilayer. Scale bar = 20 µm. (**b**) Suit2-007 cells beginning invasion of mesentery (24 h). White arrows indicate filopodia. Scale bar = 20 µm. (**c**) Top down and side view of control and G1 (1 µM) treated cells on mesenteries after 1 day. Scale bar = 10 µm. (**d**) Top down and side view of control and G1 (1 µM) treated cells on mesenteries after 10 days. Scale bar = 10 µm. (**e**) Mesh representations of invading cells after 1, 5, or 10 days from volume analysis. G1 (1 µM) + RhoA rescue mesh representation on Day 1 only. Blue mesh = cells, grey plane = top layer of mesentery. Scale bar = 2 μm. Arrow represents direction of invasion. (**f**) Quantification of cell area below top layer of mesentery for 1, 5 and 10 days. For control, n = 53, 34 and 55 cells for day 1, 5, and 10 respectively. For G1, n = 56, 39 and 27 cells for day 1, 5, and 10 respectively. For G1 + RhoA, n = 80 cells. * represents Mann–Whitney test between control and G1-treated cells for each individual time point, *** *p* < 0.001. (**g**) Cumulative count of cells invaded through mesenteries attached to bottom of well in 24 well plate for control and G1 (1 µM) treated Suit2-007 cells. Mesenteries were transferred to a new well each day, and cells attached to the bottom of the old well, where mesenteries had been for 24 h, were counted. Cell count was normalised depending on the amount of mesenteries in a well, each with the same amount of cells seeded on top of them. Each point is the sum of the mean values for each day, with standard error for each day calculated as the sum of standard errors for all the days used in summation. For Day 1-10, control = 13, 21, 23, 13, 21, 10, 17, 12, 13, 16 regions. For Day 1–10, G1 = 22, 19, 13, 11, 12, 10, 5, 5, 5, 7 regions. *p* = 0.00032 for straight line slope comparison.

Mesenteries extracted from mice have been shown to be a suitable mimic for an in vivo basement membrane [54], which cells can invade through [23]. We have previously reported a protocol for the use of mouse mesenteries to assess pancreatic cancer cell invasion through the basement membrane [55]. We extracted mesenteries from mice and confirmed their integrity and composition by staining for laminin 111 and perlecan. We observed a continuous laminin 111 network, arranged as a bilayer (Figure 6a), in concurrence with previous analysis of these mesenteries [23].

We seeded Suit2-007 cells on top of mesenteries (Supplementary Figure S12) and assessed their invasion. We fixed the cellular mesenteries at three time points (1, 5 and 10 days), and stained the mesenteries with laminin 111, while fluorescently labelled phalloidin was used to label the actin cytoskeleton of cells (Figure 6b). To quantify invasion, confocal images were captured every 0.2 µm to form a z-stack, the cell volume calculated and modelled in silico, then the percentage of the cell volume below the upper laminin layer calculated with a custom code. 

We observed that after 24 h, control cells had invaded on average 14% into the membrane, whereas G1-treated cells had invaded only 4%. We observed that when G1 was used with RhoA rescued cells, invasion at Day 1 was recovered to levels higher than control (Figure 6c–f), indicating that G1 acts by reducing contractility, in line with our previous results.

After 5 days, the difference is large between control and G1-treated cells, which had invaded 70% and 30% into the membrane respectively. By 10 days average invasion was lower, at 30% and 26% for control and G1 (Figure 6c–f), since this analysis method can only analyse those cells that have not fully invaded i.e., are still in contact with the mesentery.

To ensure that the decrease in cell invasion we saw at 10 days was due to cells having fully invaded through the membrane, we counted cells attached to the wells beneath the mesenteries. Invasion of control cells proceeded faster than G1-treated cells, indicating that GPER activation inhibits invasion (Figure 6g). 

## 3. Discussion

**Figure 7:**
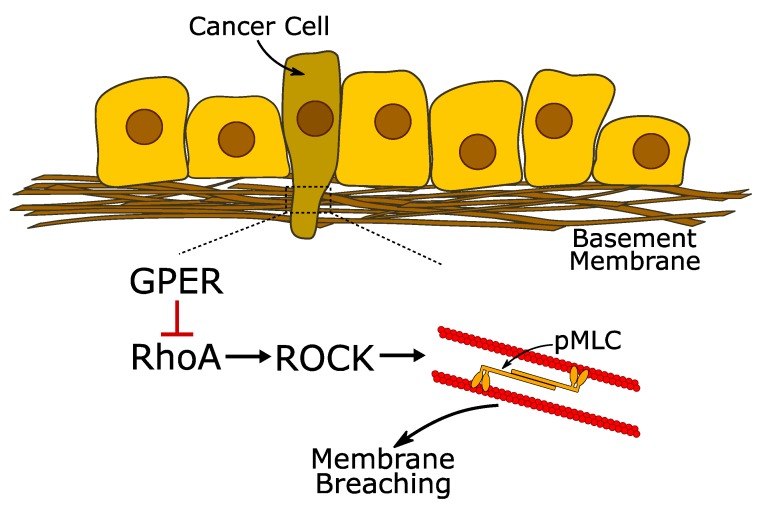
GPER inhibits membrane invasion via the RhoA/ROCK (Rho-associated protein kinase) system. The first step in the metastatic process is the invasion of neighbouring tissues, which requires the cells to breach the basement membrane. In pancreatic and prostate cancer cells, GPER can reduce their ability to breach the basement membrane by acting as a mechanoregulator. Pharmacological activation of GPER using G1 or Tamoxifen inhibits the activity of RhoA, preventing the subsequent activation of ROCK and the formation of phospho-myosin light chain 2 (pMLC-2). By controlling the activity of pMLC-2, GPER activation can modulate the contractile actomyosin machinery and regulate the mechanical activity of the cell.

Metastasis is a multi-step process [21] and cell contractility and mechanotransduction are closely associated with the invasive mesenchymal phenotype [32]. Our results indicate that activation of GPER in both pancreatic and prostate cancer cells inhibits these mechanical properties via the protein kinase A–RhoA–myosin-2 axis and concurrently inhibits pancreatic cancer cell invasion through an in vitro basement membrane (Figure 7). GPER also regulates proliferation and EMT. Our results indicate that GPER may be a pertinent target for regulating the mechanical properties of cancer cells.

In breast cancer, GPER expression has been observed as higher in ductal cells than in adjacent normal cells from the same patient [56], seemingly in contrast with our findings. Our results indicate that multiple cancer types, including breast cancer, show reduced GPER expression compared to their healthy controls. The methodology used in the previous study used only adjacent normal tissue as a control instead of healthy patients, and so is not directly comparable with our larger scale analysis. We have also shown the link between GPER and prognosis, and more recent results concur with our data demonstrating that low GPER expression correlates with lower survival in breast cancer [57].

Our observation that both cell contractility and basement membrane invasion are inhibited by GPER activation indicates that in this pancreatic cancer cell line at least, a contractile phenotype, rather than a soft and compliant phenotype, greatly enhances invasion. Additionally, G1 is seen to decrease cell rigidity in vitro to inhibit invasion. Contractility and deformability allow cells to change their migration behaviour depending on the environment [32], and the different desmoplastic stroma that exist in different organs [58] may underlie why different cell lines vary in their requirement for contractility/deformability in invasion. In the case of pancreatic cancer, we have observed that cell contractility seems to be more important for invasion than deformability.

The effect of GPER activation on cell malignancy, mechanotransduction and cell contractility is unlikely to be limited to these particular cell lines. For cell lines from the independent organs of pancreas and prostate, where metastasis from one to the other is extremely rare [59], the same effects of GPER activation on cell mechanical properties were seen, indicating that the effects are not specific to one organ. For example, YAP is essential in mechanotransduction [60], and our observation that G1 can modulate YAP activation in a RhoA-dependent manner suggests the wide ranging mechanical relevance of GPER. RhoA, in turn, orchestrates the mechanical activity of the cell, and its activation is regulated by a myriad of processes [61], including more recently reported mechanotransduction mechanisms such as the protein unfolding-induced activation of upstream effectors [62].

The composition of the epithelial basement membrane has been shown to facilitate invasion in many cancers [21,26,27], and our analysis of pancreatic basement membranes has revealed changes in the expression of laminins, a disorganised arrangement of collagen IV in the progression of PDAC, and the presence of a bilayer in pancreatic tissue. Our observation that GPER activation inhibits invasion through a laminin bilayer structure is highly relevant due to the increased laminin seen in PDAC. Laminin is closely linked to mechanotransduction, as cell-laminin interactions proceed through integrins [63], mechanosensitive molecules that link directly to the actomyosin cytoskeleton [64]. Cells have been shown to remodel the basement membrane through traction forces in an integrin-dependent manner [65], and this correlates with the disordered organisation of collagen IV we observe in PDAC. In recent years, advances in the field of mechanobiology have highlighted the need to recapitulate the mechanical complexity of the cellular microenvironment [66]. The basement membranes mimics used here illustrate the potential of these platforms as a tool to study complex biomechanical processes such as cancer cell invasion. 

We present GPER as a critical signalling hub linking cell mechanics and malignant properties such as proliferation and invasion. GPER is associated with the presence of multiple cancers, and also the survival of patients with these cancer types, confirming the necessity of investigation into its cellular effects. Here, we observe two independent cell lines to be mechanically affected by GPER activation, and since there are limited studies linking GPER to mechanical properties, future studies that correlate GPER activation with cell malignancy and mechanical properties may shed light on the reasons why cells respond differently to GPER activation. Moreover, because GPER can be activated by tamoxifen, a drug that has been widely used to treat breast cancer for the last two decades, our work offers the possibility of repurposing tamoxifen to mechanically reprogram pancreatic and prostate cancer cells. The development of new techniques to analyse the mechanical behaviour and properties of cells [67] will enable more advanced studies on the cellular effects of GPER signalling in the future.

## 4. Materials and Methods 

### 4.1. TCGA Database

RNA-seq data from cancer types was downloaded from the TCGA cBioPortal (http://www.cbioportal.org/index.do) in Nov. 2017 and Feb. 2018. Gene abundance was quantified as RSEM (RNA-seq by Expectation Maximisation) [28]. Statistical analysis was performed with the Wilcoxon rank-sum test. 

### 4.2. Cell Culture

Suit2-007 cells were cultured in DMEM medium with 10% FBS (Cat No.F7524, Sigma Aldrich, Dorset, UK), l-glutamine (Cat No.G7513, Sigma Aldrich), penicillin/streptomycin (Cat No.P4333, Sigma Aldrich), and sodium pyruvate (Cat No.11360-039, Gibco, Carlsbad, CA, USA). PC-3 cells were cultured in RPMI 1640 medium (Cat No.R8758, Sigma Aldrich) with 10% FBS (Cat No.F7524, Sigma Aldrich), l-glutamine (Cat No.G7513, Sigma Aldrich), penicillin/streptomycin (Cat No.P4333, Sigma Aldrich), sodium pyruvate (Cat No.11360-039, Gibco, Carlsbad, CA, USA). Coverslips (Cat No.631-0149P, cover glasses, 13 mm diameter, thickness No.1, VWR, Radnor, PA, USA) were incubated with 10 µL/mL of Fibronectin (Cat No.PHE0023, Gibco, Carlsbad, CA, USA) in PBS (Cat No.D8537, Sigma Aldrich) for 45 min at 37 °C. Cells were collected and counted using a haemocytometer and seeded on the activated coverslips.

For tamoxifen ((Z)-4-Hydroxytamoxifen, Cat No.H7904-5MG, Sigma Aldrich), G1 (Cat No.3577, Tocris, Bristol, UK) or GPER antagonist (G15; Cat. No.3678, Tocris) treatment, cells were left to attach to fibronectin-coated coverslips for at least 6 h, and media was changed for media not containing phenol red. The next day, tamoxifen/G1/GPER antagonist at the required concentration in clear media was given to the cells and cells were left to incubate for the desired time period.

For preparation of plasmids for transfections, lyophilised GPER siRNA plasmids (Santa Cruz Biotechnology, sc-60743, Dallas, TX, USA) were resuspended in 330 μL of RNase-free water to make a 10 μM solution. The constitutively active RhoA plasmid (pRK5-myc-RhoA-Q63L) was a gift from Gary Bokoch (Addgene plasmid # 12964). This plasmid was used as a template to create the plasmid RhoA (S188A/Q63L) by substitution of the serine amino acid in position 188 to alanine using site directed mutagenesis. The final concentration of purified RhoA plasmids was 3126 ng/μL. The plasmids were stored at −20 °C.

In total, 80%–90% confluent cells were transfected with GPER siRNA plasmids or RhoA rescue plasmids by electroporation using the Neon Transfection System (Thermo Fisher, Carlsbad, CA, USA) according to manufacturer’s instructions with one pulse of 1300 V for 30 ms. After transfection, the cells were recovered in clear medium without antibiotics for 24 h. The medium was then replaced with more clear medium, and the transfected cells were incubated for another 24 h. All the measurements of the transfected cells were conducted 2 days after transfection. 

### 4.3. Human Tissue Immunofluorescence

For basement membrane staining, sections were washed in PBS for 10 min, treated with 0.2% Triton X-100 + 0.5% formaldehyde in PBS for 5 min, washed for 15 min with PBS, fixed for 20 min with 4% formaldehyde, then washed for 15 min in PBS + 1% BSA. Primary antibodies were diluted 1:100 in PBS + 1% BSA and incubated with sections overnight at 4 degrees. Sections were then washed for 1 h in PBS + 1% BSA, treated with secondary antibodies (1:100 in PBS + 1% BSA) + DAPI (1 in 10,000) for 2 h, washed for 1 h in PBS + 1% BSA, then mounted in Vectashield (Vector Laboratories, Burlingame, CA, USA).

### 4.4. In Vitro Immunofluorescence

Cells on coverslips were washed twice with PBS, then fixed with 4% paraformaldehyde for 10 min at 37 °C. Coverslips were washed with PBS then permeabilised for 5 min with 0.1% Triton X-100 in 2% BSA/PBS. Cells were then further blocked for 30 min with 2% BSA/PBS. After 2 washes with PBS, cells were incubated with primary antibody (1 in 200) for 1 h, washed twice with PBS, then incubated with secondary antibody (A11034, Alexa Fluor^®^ 546, Thermo Fisher) (1 in 200) and phalloidin (A22283, Alexa Fluor^®^ 546, Thermo Fisher) (1 in 500) for 1 h in the dark. After a final 2 washes, sections were mounted in ProLong Gold with DAPI (P36935, Thermo Fisher) and imaged with a Nikon Ti-Eclipse (Ti Eclipse; Nikon, Tokyo, Japan) microscope with a 40× objective. Immunofluorescence intensity was calculated as the mean value of marker intensity in the selected region minus the mean value of a background region. 

### 4.5. Quantitative PCR

Total RNA was extracted using the RNeasy Mini kit (74104, Qiagen, Hilden, Germany) and 1 μg of total RNA was reverse-transcribed using the High-Capacity RNA-to-cDNA kit (4387406, Thermo Fisher) according to the manufacturer’s instructions. qPCR was performed using the SYBR Green PCR Master Mix (4309155, Thermo Fisher) with 100 ng cDNA input in 20 μL reaction volume. RPLP0 expression level was used for normalisation as a housekeeping gene. The primer sequences for were as follows: RPLP0 forward 5′-CGGTTTCTGATTGGCTAC-3′ and reverse 5′-ACGATGTCACTTCCACG-3′, Vimentin: forward-5′-GGAAACTAATCTGGATTCA-3′, reverse-5′-CATCTCTAGTTTCAACCGTC-3′; E-cadherin: forward-5′-CCGAGAGCTACACGTTC-3′, reverse-5′-TCTTCAAAATTCACTCTGCC-3′, CTGF: forward 5′-TTAAGAAGGGCAAAAAGTGC-3′, and reverse 5′-CATACTCCACAGAATTTAGCTC-3′, ANKDR1: forward 5′-TGAGTATAAACGGACAGCTC-3′ and reverse 5′-TATCACGGAATTCGATCTGG-3′. All primers were used at 300 nM final concentration. The relative gene expression was analysed by comparative 2−ΔΔct method.

### 4.6. Western Blots

Cells were lysed in RIPA buffer supplemented with Halt protease and phosphatase inhibitor cocktail (both Thermo Fisher). Lysates were kept on ice with periodic agitation for 30 min, followed by centrifugation at 16,000 *g* for 20 min. The insoluble pellet was discarded, and protein concentration in the lysate measured using the BCA assay according to the kit instructions (Pierce, Thermo Fisher). Lysate was mixed with 4× Laemmli sample buffer including β-mercaptoethanol, heated at 95 °C for 5 min, and 20 μg loaded into each well of a 4%–20% gel (Mini-PROTEAN TGX, Bio-Rad, Watford, UK). Separated proteins were transferred onto nitrocellulose membrane (Bio-Rad). Total protein was quantified using REVERT total protein stain (Li-Cor, Lincoln, NE, USA), and the blot imaged using an Odyssey infrared imager (Li-Cor). Following removal of total protein stain using REVERT Reversal Solution (Li-Cor), blots were blocked in Odyssey Blocking Buffer in Tris buffered saline (TBS) (Li-Cor) for 1 h. Blots were incubated with primary antibodies diluted in TBS-tween 20 (TBST) at 4 °C overnight as follows: mouse anti-β-actin (Abcam ab8226, Cambridge, UK) at 1:10,000, rabbit anti-Rho-A,-B,-C (Merck Millipore 04-822, Burlington, MA, USA) at 1:1000, rabbit anti-pRhoA Ser188 (Abcam ab41435) at 1:1000, rabbit anti-GPER (Abcam ab39742) at 1:1000 or rabbit anti-GPER (Abcam 154069) at 1:1000. Following washes in TBST, blots were incubated with secondary antibodies in TBS-T for 1 h follows: IRDye 680RD donkey anti-mouse IgG (H+L) (Li-Cor 925-68072) at 1:15,000 or IRDye 800CW donkey anti-rabbit IgG (H+L) (Li-Cor, 925-32213). Following washes in TBST, blots were imaged using an Odyssey infrared imager. Quantification of protein bands and total protein lanes was carried out using Image Studio Lite 5.2 (Li-Cor).

### 4.7. cAMP Assay

Suit2-007 cells were seeded in a 96-well plate. Then, 24 h after seeding, they were incubated with 100 μM IBMX (3-Isobutyl-1-methylxanthine; Sigma-Aldrich) for 20 min, followed by incubation with 10 μM forskolin (sc-3562; Santa Cruz Biotechnology) and 100 μM IBMX or 10 μM forskolin, 100 μM IBMX and 1 µM G1 GPER agonist for a further 20 min. Cyclic AMP levels were measured using cAMP-Glo kit (V1501, Promega, Madison, WI, USA) based on an inversely proportional bioluminescent luciferase reaction. Cyclic AMP levels are represented as inversed luminescence values normalised to control conditions (IBMX + forskolin). Forskolin was used to increase the baseline cAMP levels past the assay threshold and to potentiate the receptor to more accurately determine the response to the agonist as described in [37].

### 4.8. Magnetic Tweezers

To assess how cancer cells sense and respond to applied forces, we use a magnetic tweezers protocol previously developed by our group and others [68,69]. Paramagnetic microbeads with a diameter of 4.5 μm (Dynabeads M-450 Epoxy; Thermo Fisher) were coated with fibronectin (Thermo Fisher) according to the manufacturer’s instructions. The fibronectin-coated beads were incubated with cells for 30 min at 37 °C to enable attachment to the surface integrins. Cells were briefly washed with PBS to remove unbound beads prior to analysis. 

Individual magnetic beads attached to cells were subjected to a pulsatile force regimen applied with a custom-built magnetic tweezer apparatus consisting of an electromagnet and an inner core. The pulsatile force regime consisted of 12 pulses, with a force amplitude of 1 nN, a duration of 3 s per pulse and a period of rest between each pulse of 4 s. The displacement of the beads in response to the 12 force pulses was recorded with an inverted microscope (Ti Eclipse; Nikon) and analysed using a custom MATLAB script. 

The ability of the cells to sense and respond to the applied tension (mechanosensing) was quantified by the relative decrease in the amplitude of the displacement of the bead i.e., in cells with high mechanosensing activity, the relative bead displacement decreases over the 12 pulses as the cell stiffens in response to the applied force. 

### 4.9. Cell Compliance

Cells were seeded and treated on fluorodishes. Cell compliance measurements were conducted on a JPK Nanowizard-1 (JPK Instruments, Berlin, Germany) operating in force spectroscopy mode, mounted on an inverted optical microscope (IX-81; Olympus, Shinjuku-ku, Japan). AFM pyramidal cantilevers (MLC-2T; Bruker, Billerica, MA, USA) with a spring constant of 0.03 N/m were used with a 15 μm diameter glass bead attached to cantilever tip. Prior to measurements with the adapted cantilevers, their sensitivity was calculated by measuring the slope of force-distance curve in the AFM software on an empty region of the petri dish. For indentation tests, the cantilever was aligned over the cell away from the nucleus, and for each dish, 30 force curves were acquired across 30 cells. Force-curve acquisition was carried out with an approach speed of 5 μm/s and a maximum set force of 1 nN. Elastic moduli were calculated from the force-distance curves by fitting the contact region of the approach curve with the Hertz contact model [70] using the AFM software (JPK).

### 4.10. Elastic Pillars

Elastic micropillars were fabricated in PDMS via replica moulding as described elsewhere [69]. Briefly, PDMS (Sylgard 284, Corning, Midland, MI, USA) was mixed at a 10:1 ratio of prepolymer to curing agent, degassed and poured on an etched silicon mould. PDMS was cured at 80 °C for 12 h, resulting in an elastic modulus of 2 MPa. Based on this elastic modulus and the dimensions of the pillars (5 μm height, 1 μm diameter), the pillar spring constant was calculated to be 2.35 nN/μm.

Pillar arrays were coated with human plasma fibronectin (10 mg/mL; Sigma-Aldrich), incubated at 37 °C for 1 h before measurements, then excess fibronectin washed away with PBS. Cells were trypsinised from culture flasks, suspended in media, and seeded onto the pillars. Cells were then left to attach for 1 h, Bright-field time-lapse imaging of the pillars was conducted with an inverted microscope (Ti Eclipse; Nikon) with the samples held at an ambient temperature of 37 °C. Image sequences were recorded with a sCMOS camera (Neo sCMOS; Andor, Belfast, UK) every 1 s for 1 min using a 40× (0.6 NA, air; Nikon) objective. Each dish was analysed for a maximum of 30 min. The position of each pillar in the time-lapse videos was tracked using a custom MATLAB program to track the centre of a point spread function of the intensity of the pillars across all frames. By selecting a location free of cells, tracking of a small set of pillars allowed a measurement of the stage drift to be obtained and corrected for in the data set. The time-dependent displacement of a given pillar was obtained by subtracting the initial position of the pillar (zero force) from the position in a given frame. Traction forces were obtained by multiplying the pillar displacements by the pillar stiffness—the maxima for each pillar were found to obtain the peak forces across the cell.

### 4.11. Transwell Invasion Assay

The Transwell (CLS3422, Corning, Tewksbury, MI, USA) invasion assay was performed as described with 8 µm diameter pores [71]. In total, 100 µL of 0.1 mg/mL collagen I (354236, BD Biosciences, Bedford, MA, USA) solution in PBS was added to the top of the Transwell and left to dry for 1 h at 37 °C. Serum-containing media was placed in a well of a 24 well plate and the Transwell added into the well. Cells were counted and resuspended in serum-free medium, and 100,000 cells added to each well.

Then, 24 h later, Transwells were washed, and a cotton-tipped applicator was used to rub cells off the top layer (where they were initially seeded). Transwells were then fixed with 70% ethanol solution for 10 min and washed twice with PBS. Transwells were then stained in 0.1% crystal violet solution, then washed 3× with PBS before being left to dry and imaged.

### 4.12. Mesentery Invasion Assay

Mesenteries were extracted from mice, and attached using Vetbond (1469SB, 3M, St Paul, MN, USA) to Eppendorf tubes, cut to approximately 1 cm height, and stored in PBS + NaN_3_. Before cell seeding, mesenteries were transferred to wells containing 1 M ammonium hydroxide for decellularisation for 1 h, then washed with PBS. Mesenteries were then placed in serum-containing medium, and cells were collected and resuspended in serum-free medium before seeding on top of mesenteries. Cells were left for 1, 5, or 10 days to invade, then fixed with 4% paraformaldehyde. Once washed by PBS, mesenteries were treated with 0.5% Triton-X for 5 min, washed with PBS, blocked with 2% BSA/PBS for 30 min, washed with PBS, treated with primary antibodies (1 in 100) in 2% BSA/PBS for 1 h at room temperature, washed with PBS, treated with secondary antibodies (1 in 200) in 2% BSA/PBS for 1 h at room temperature in the dark, washed with PBS, then mounted in ProLong AntiFade Gold with DAPI (P36935, Thermo Fisher) overnight at 25 °C.

### 4.13. Quantification of Percentage Volume Invasion of Basement Membrane

In order to assess membrane invasion, we analysed mesenteries using confocal fluorescence microscopy (Ti Eclipse; Nikon). For each mesentery, an average of 5 randomly selected fields of view were analysed, with an average of 10 cells per field of view. All experiments were conducted in triplicate.

To quantify invasion of the basement membrane, we developed a novel Python-based algorithm, Quantification of Percentage Invasion (Q-Pi). Q-Pi employs a series of filters to recognize cell edges and fit regression ellipses using the Teh-Chin89 chain approximation algorithm [72]. The Convex Hull algorithm [73] then allows for 3D reconstruction and based on the location of the membrane on the Z axis, percentage volume of cell below the membrane can be calculated. The open source program was developed in collaboration with Upamanyu Ghose, Manipal Institute of Technology, India and can be found at https://github.com/titoghose/Q-Pi.

For the cumulative cell count, each day, we transferred each mesentery to a new well and then counted the cells that had attached to the well beneath during the previous 24 h. Multiple regions of interest were analysed for each well, and the average value for the amount of cells in each region of interest was normalised by dividing by the number of mesenteries present in that well. These average values, including their errors, were cumulated across the 10 days, giving the parameter “cumulative cells per mesentery per ROI”.

## 5. Conclusions

In this study, we characterised epithelial basement membranes from healthy donors and PDAC patients and demonstrated that they are formed by an asymmetric bilayer with different composition of laminin 111 and collagen IV. Based on these findings, we developed a method to assess cancer cell invasion using mouse mesenteries as basement membrane mimics due to their structural similarity. Using this technique, we demonstrated that activation of the G protein-coupled oestrogen receptor (GPER) inactivates the actomyosin machinery in cancer cells and inhibits their ability to invade through these membranes. 

The method presented here can be extended to other cells types and could provide a new technique to analyse cancer cell invasion and transmigration with a more biologically relevant setup that mimics the bilayer structure of the basement membrane.

## Supplementary Materials

The following are available online at https://www.mdpi.com/2072-6694/12/2/289/s1. Figure S1: Full western blot images for GPER and total protein in Suit2-007 and HEK293 cells, Figure S2: G1 only affects cell viability and proliferation after 72 h in Suit2-007 and PC-3 cells, Figure S3: Knockdown efficiency of siRNA GPER (GPER knocked down/GPER KD) in Suit2-007, Figure S4: GPER activation by G1 increases intracellular cAMP levels, Figure S5: GPER activation inhibits mechanotransduction in PC-3 cells, Figure S6: GPER activation inhibits force generation and cell compliance in PC-3 cells, Figure S7: Transfection with scramble siRNA does not affect the mechanical activity of Suit2-007 cells, Figure S8: GPER induced inhibition of EMT in PC-3 cells, Figure S9: GPER induced inhibition of EMT requires RhoA activity, Figure S10: Tamoxifen influences cell viability and proliferation at >5 μM concentration in Suit2-007 and PC-3 cells, Figure S11: Tamoxifen acts through GPER to inhibit EMT in PC-3 cell, and Figure S12: Schematic for mesentery invasion assay.
