# Supplementary figures and images for: GPER Activation Inhibits Cancer Cell Mechanotransduction and Basement Membrane Invasion via RhoA
Source: Cancers (Basel). 2020 Jan 25;12(2):289. doi: 10.3390/cancers12020289 (PMC7073197; doi:10.3390/cancers12020289)

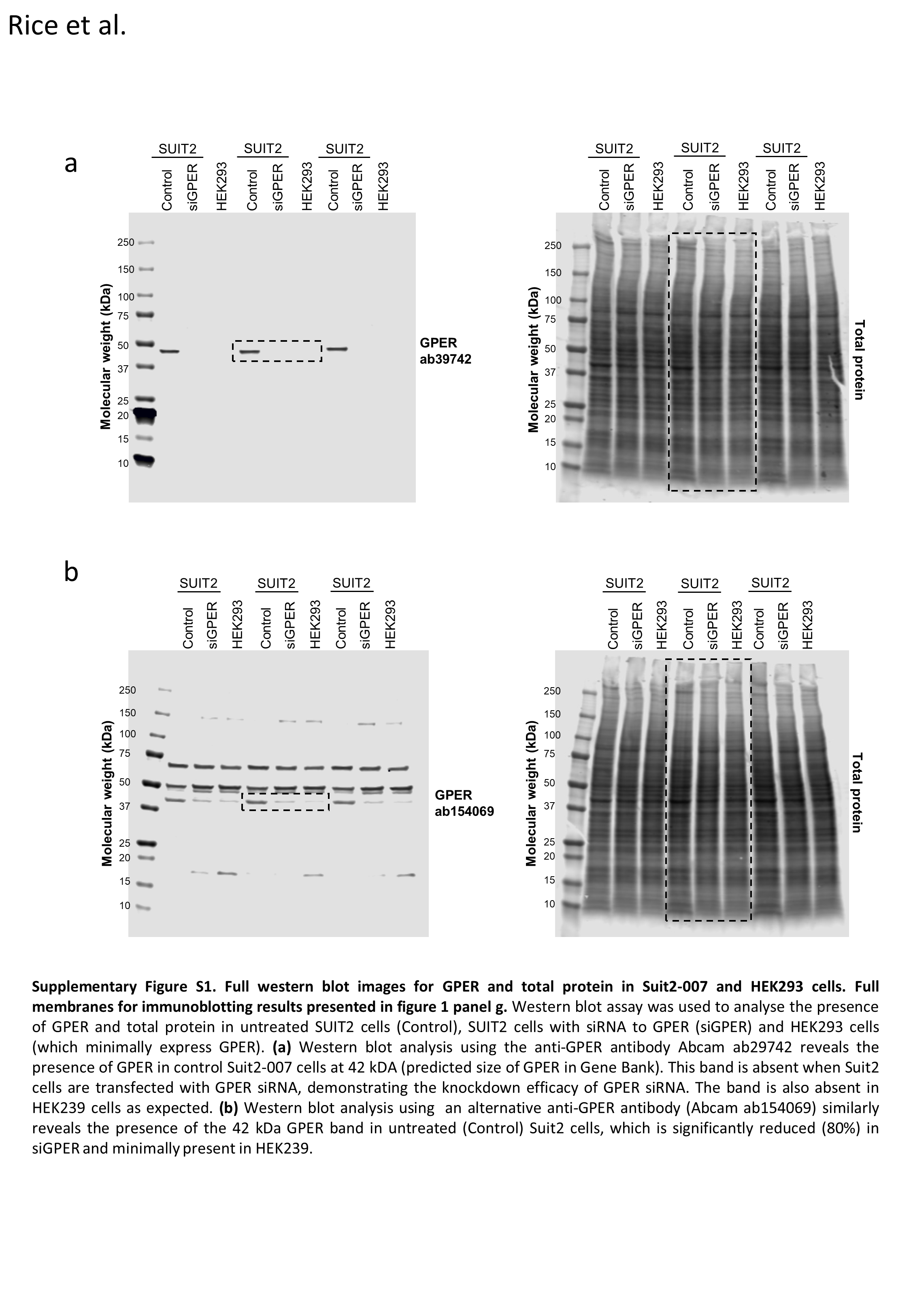

Supplement: Supplementary file 1 [file cancers-12-00289-s001.zip › cancers-679112-supplement/Supplementary Figure S1.TIF]

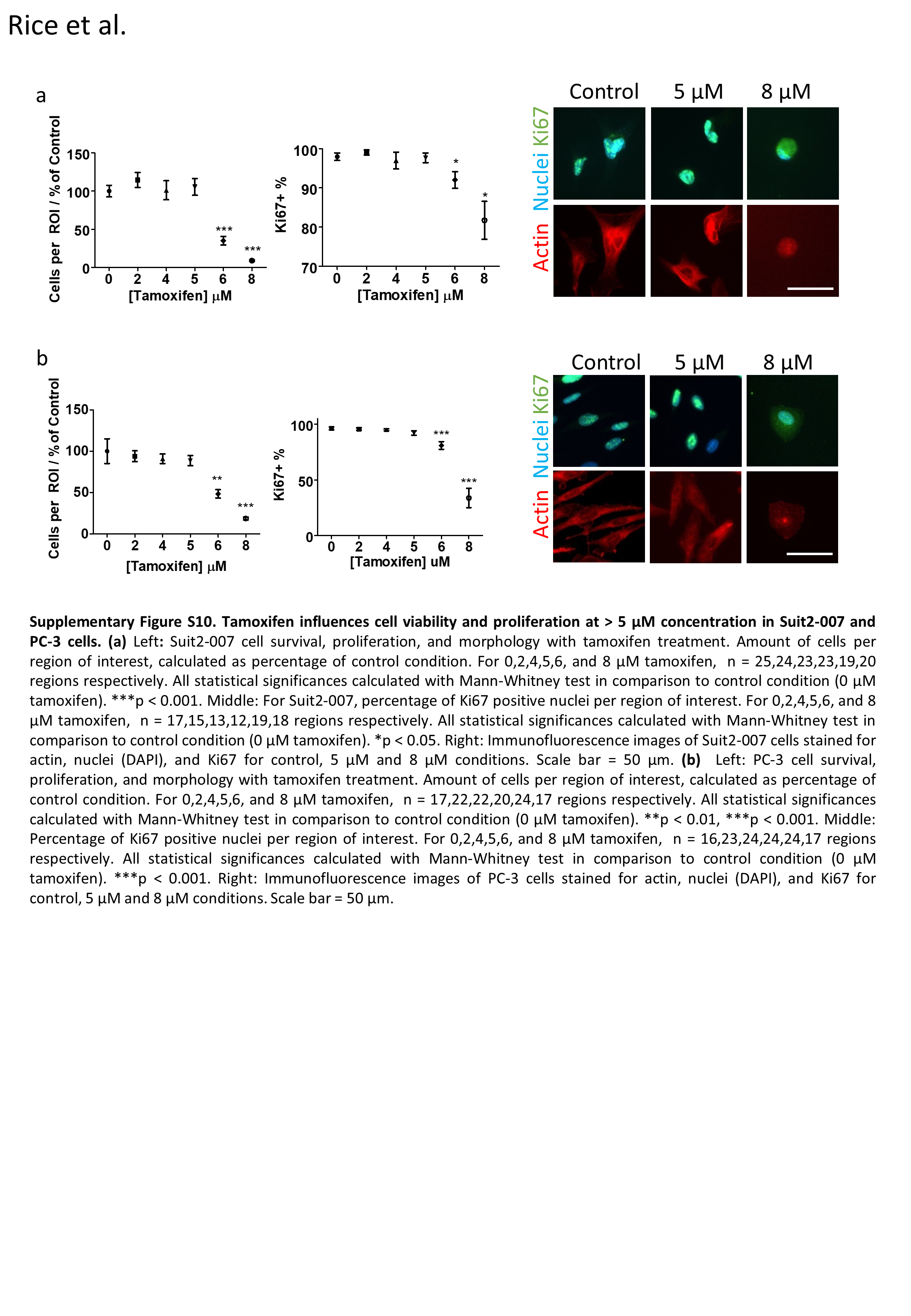

Supplement: Supplementary file 1 [file cancers-12-00289-s001.zip › cancers-679112-supplement/Supplementary Figure S10.TIF]

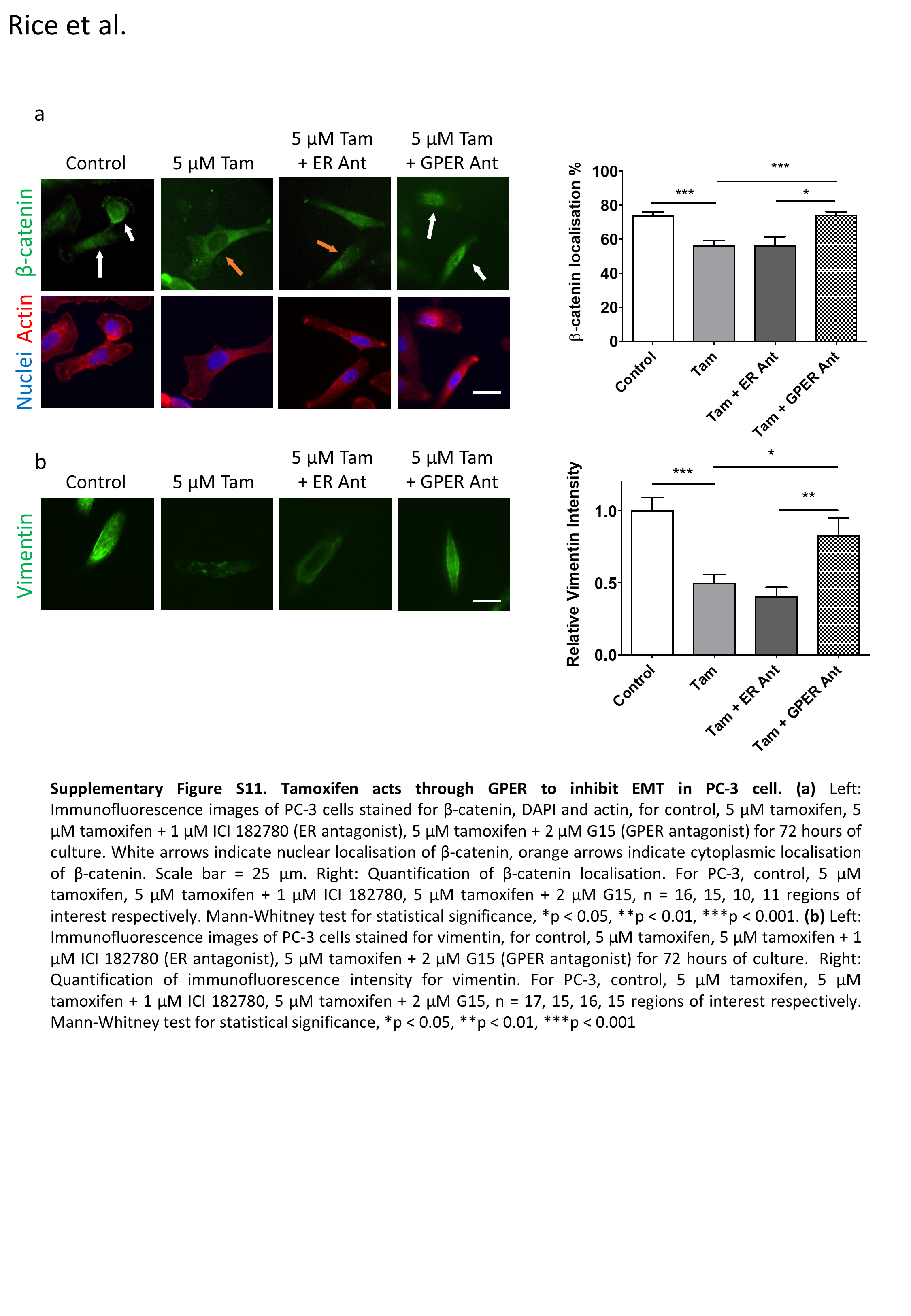

Supplement: Supplementary file 1 [file cancers-12-00289-s001.zip › cancers-679112-supplement/Supplementary Figure S11.TIF]

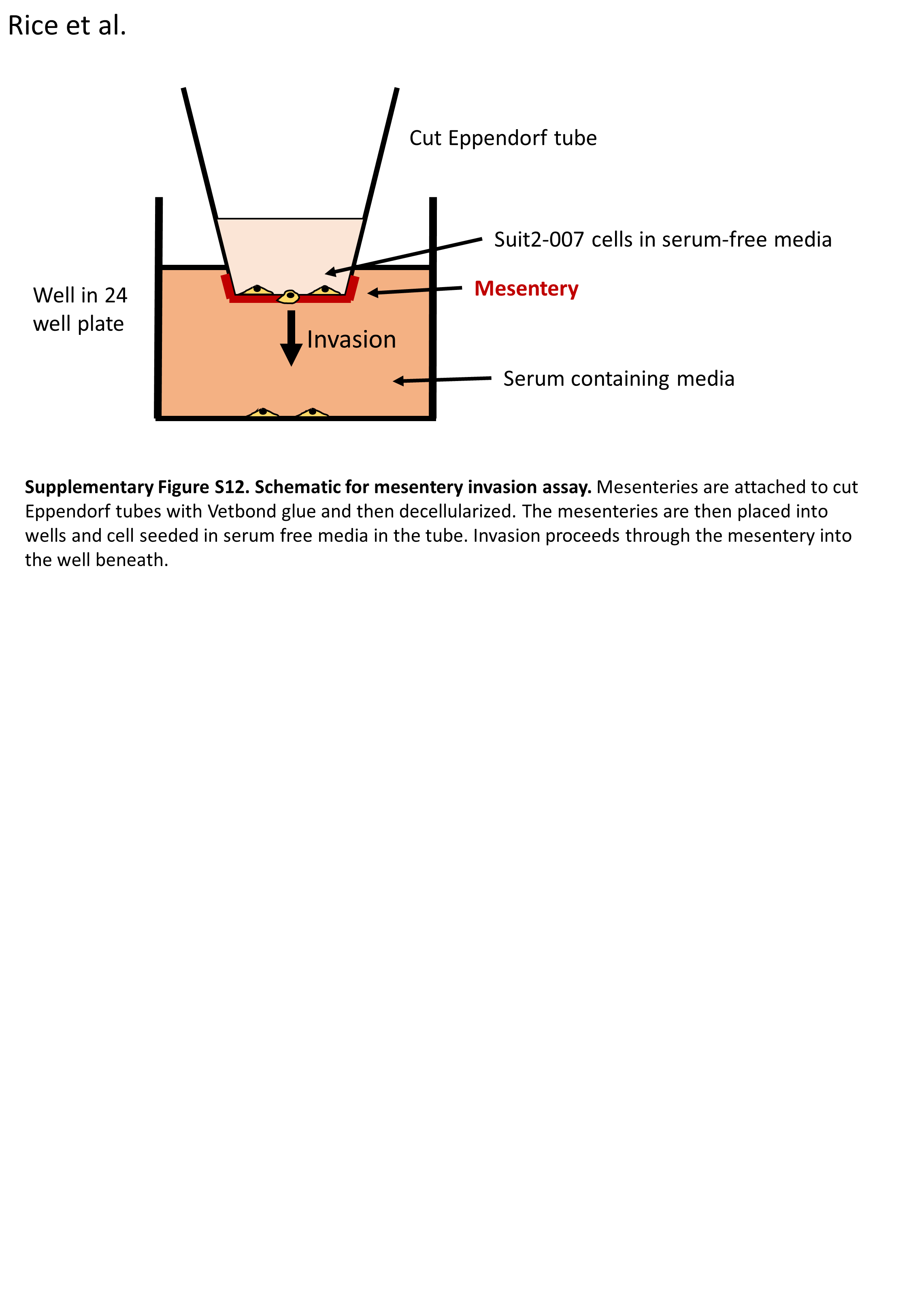

Supplement: Supplementary file 1 [file cancers-12-00289-s001.zip › cancers-679112-supplement/Supplementary Figure S12.TIF]

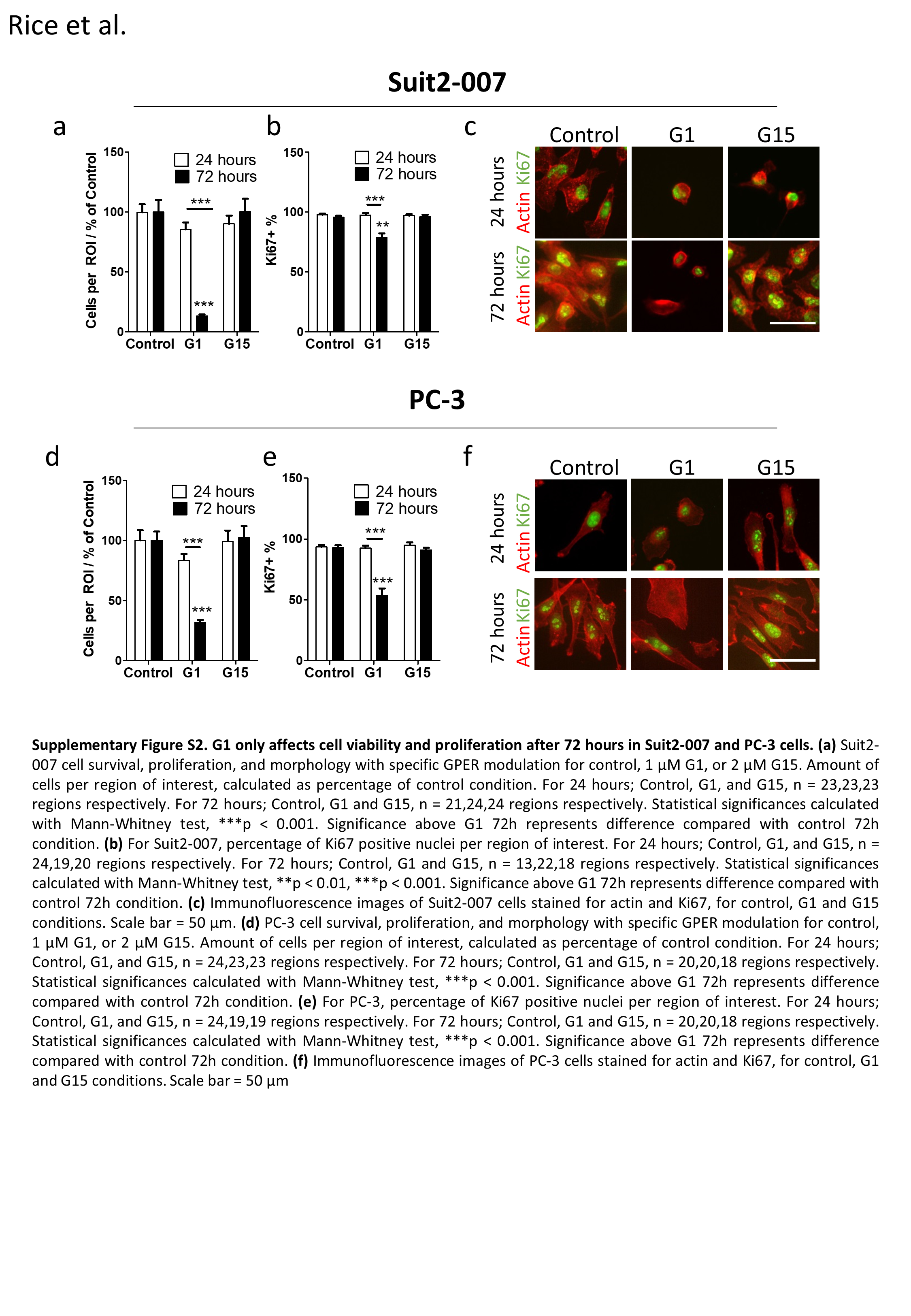

Supplement: Supplementary file 1 [file cancers-12-00289-s001.zip › cancers-679112-supplement/Supplementary Figure S2.TIF]

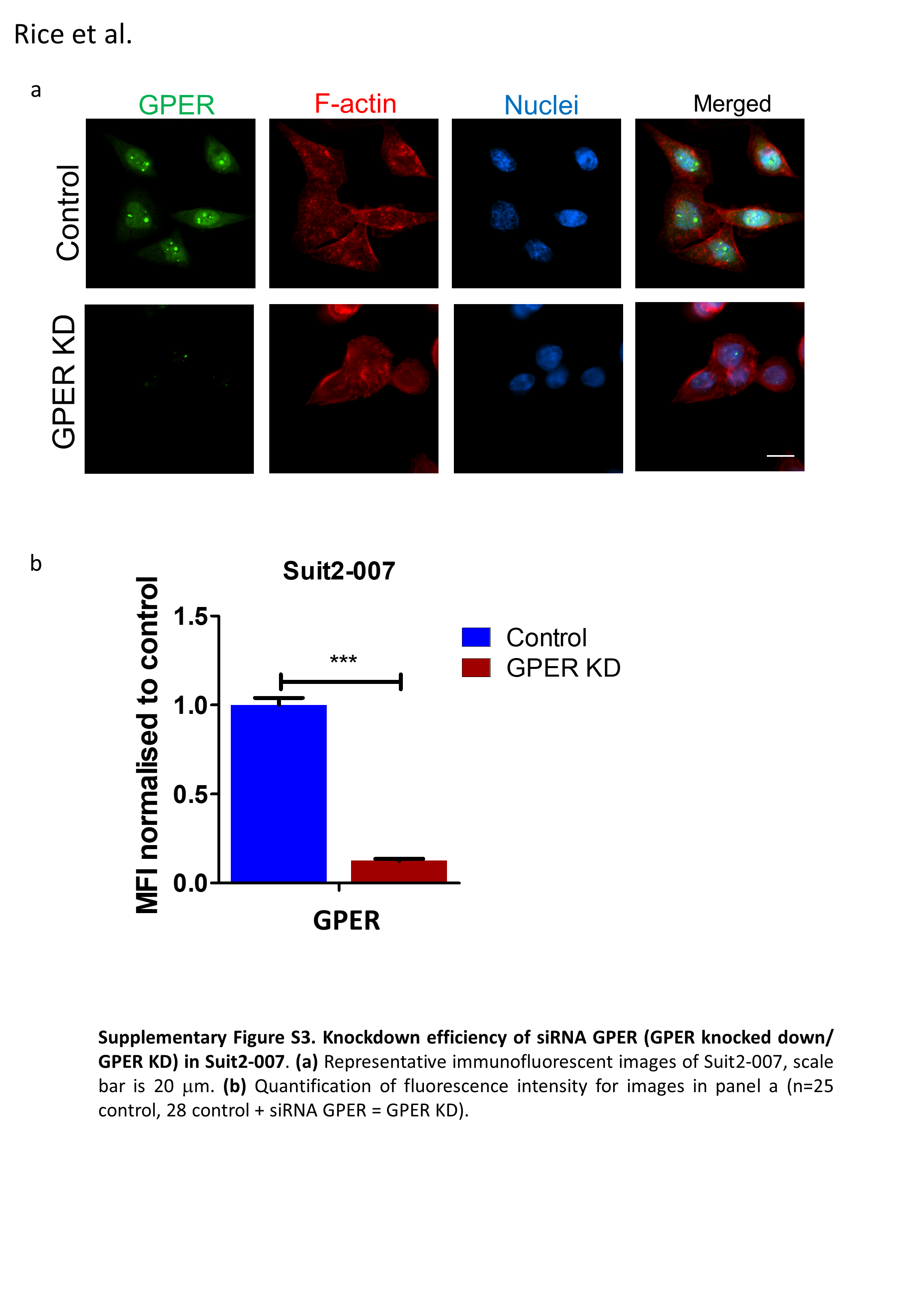

Supplement: Supplementary file 1 [file cancers-12-00289-s001.zip › cancers-679112-supplement/Supplementary Figure S3.TIF]

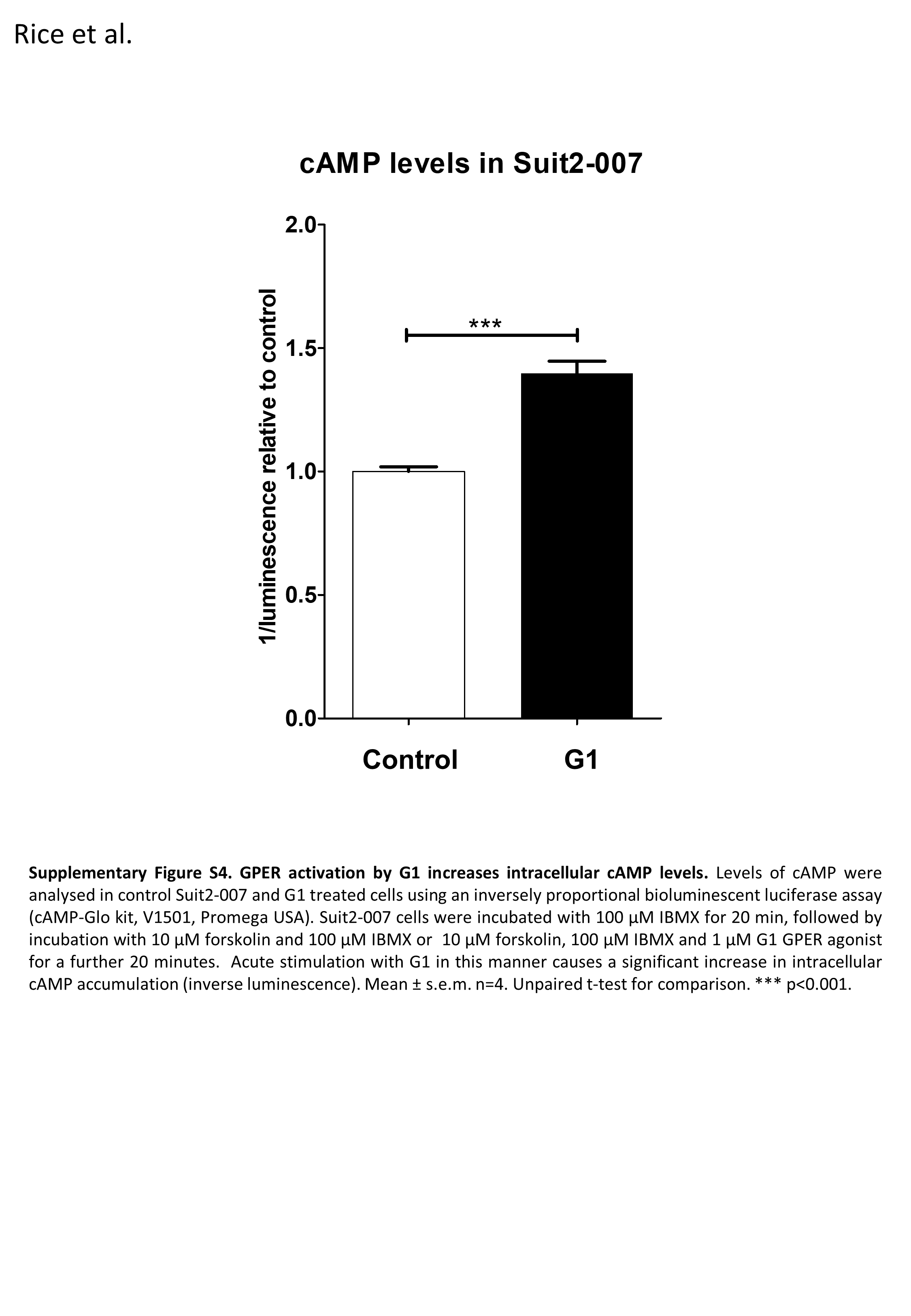

Supplement: Supplementary file 1 [file cancers-12-00289-s001.zip › cancers-679112-supplement/Supplementary Figure S4.TIF]

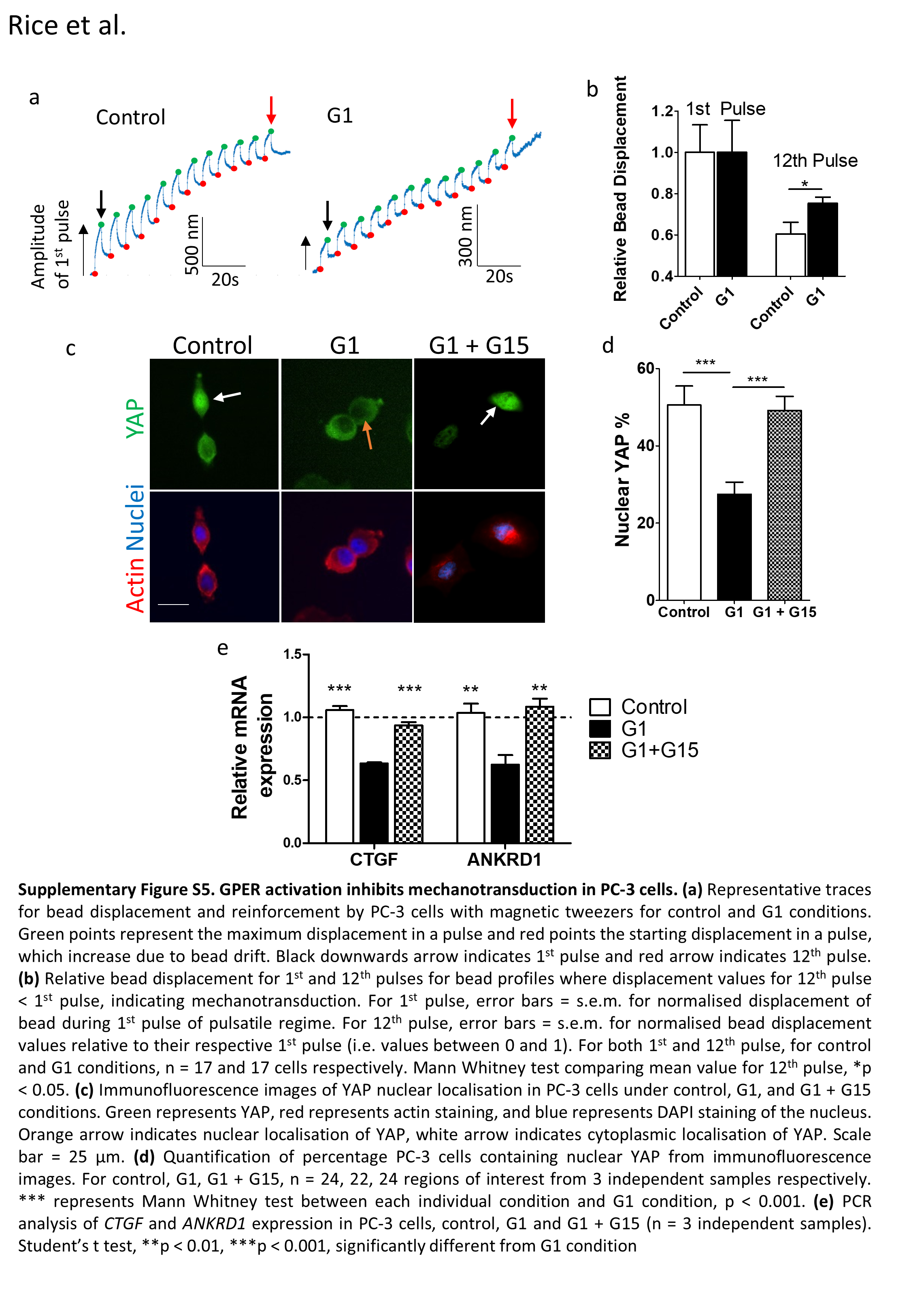

Supplement: Supplementary file 1 [file cancers-12-00289-s001.zip › cancers-679112-supplement/Supplementary Figure S5.TIF]

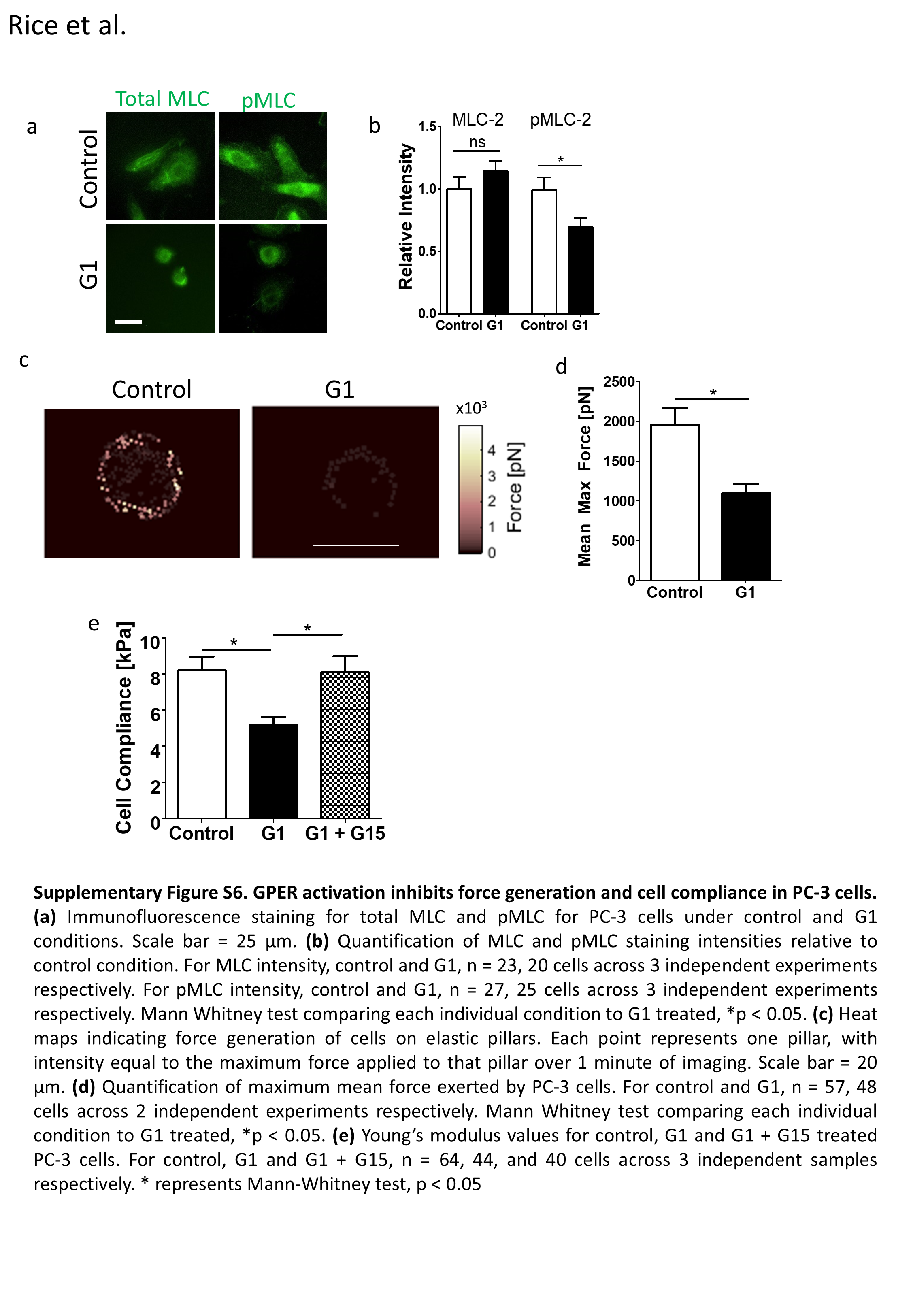

Supplement: Supplementary file 1 [file cancers-12-00289-s001.zip › cancers-679112-supplement/Supplementary Figure S6.TIF]

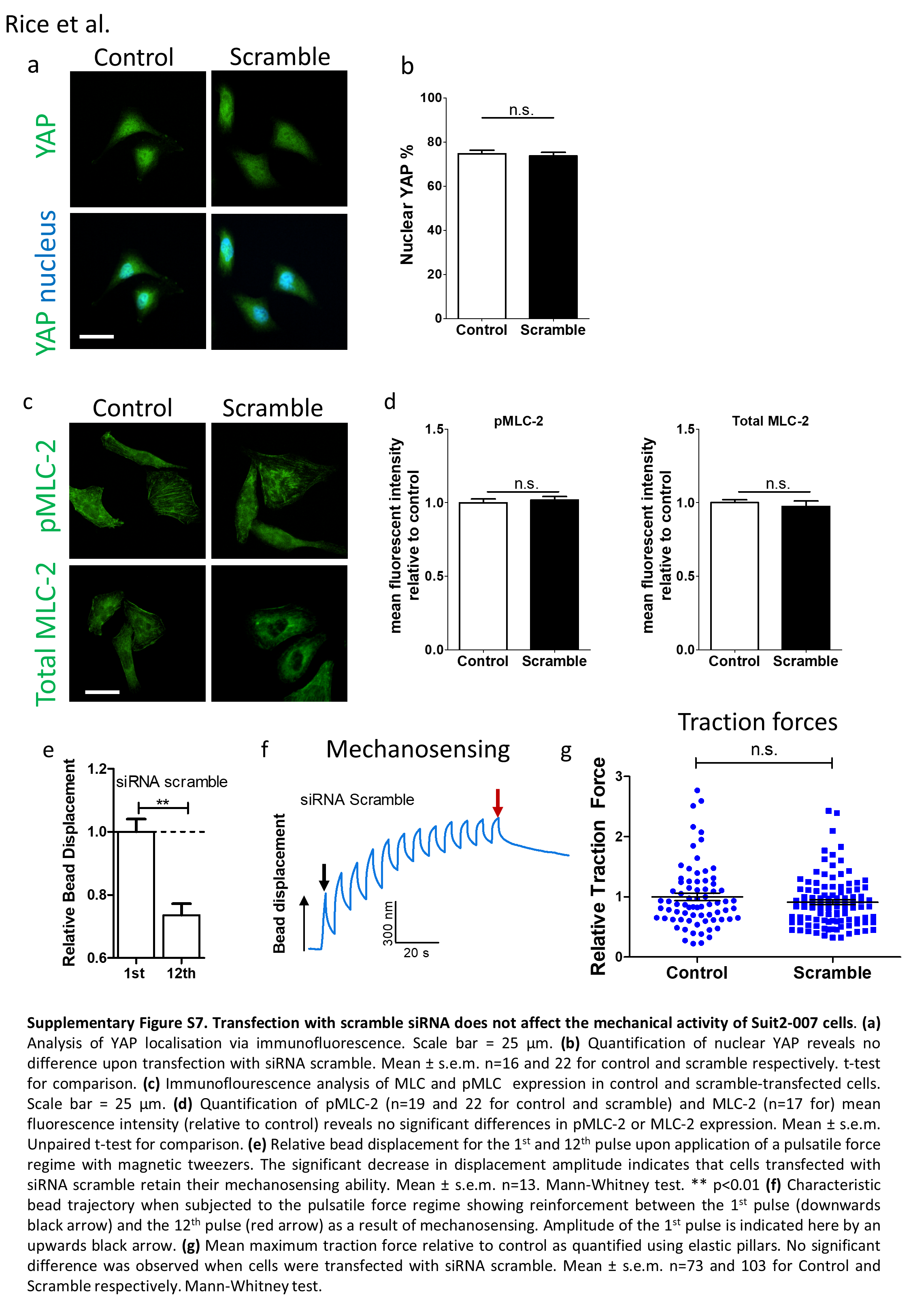

Supplement: Supplementary file 1 [file cancers-12-00289-s001.zip › cancers-679112-supplement/Supplementary Figure S7.TIF]

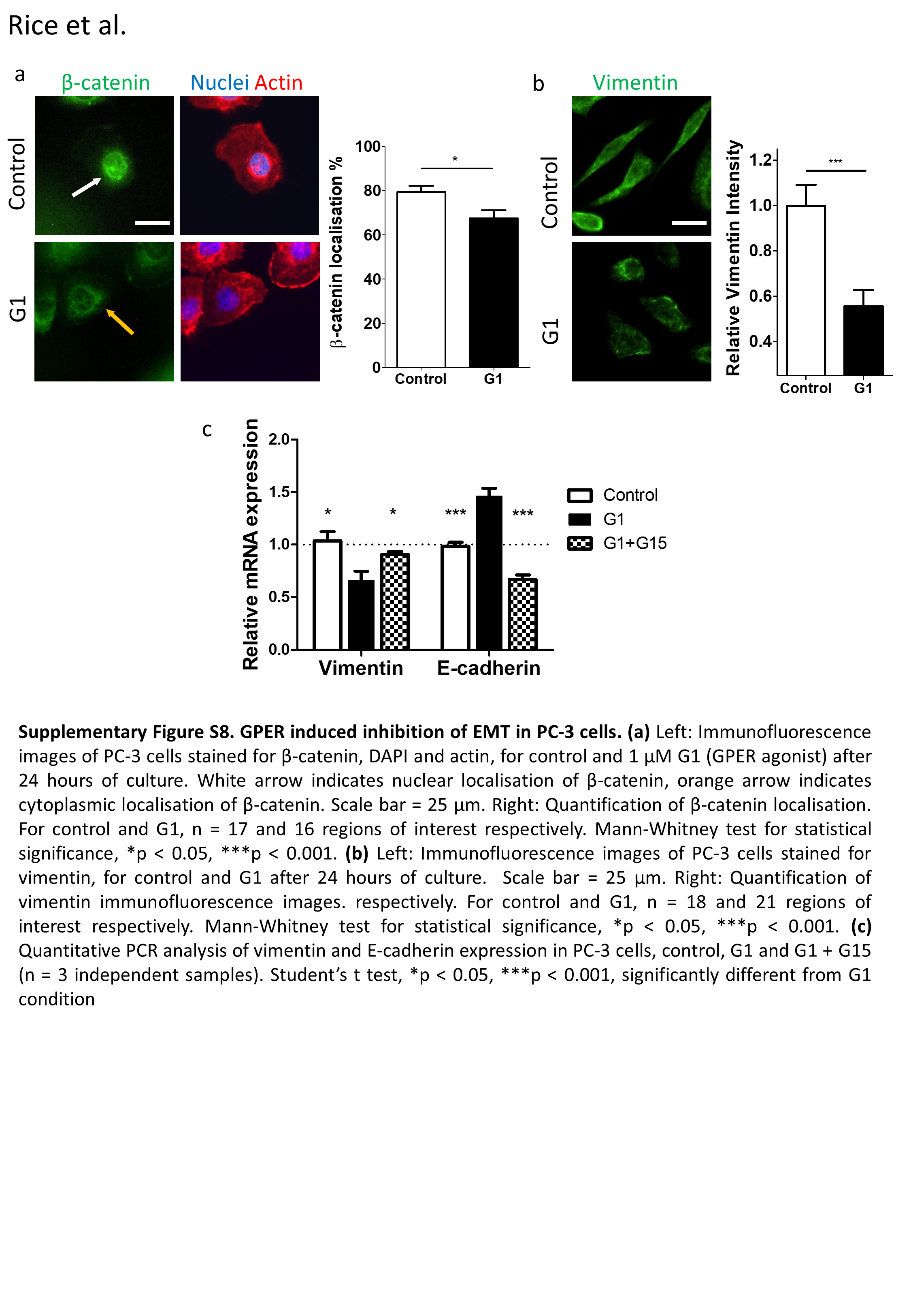

Supplement: Supplementary file 1 [file cancers-12-00289-s001.zip › cancers-679112-supplement/Supplementary Figure S8.TIF]

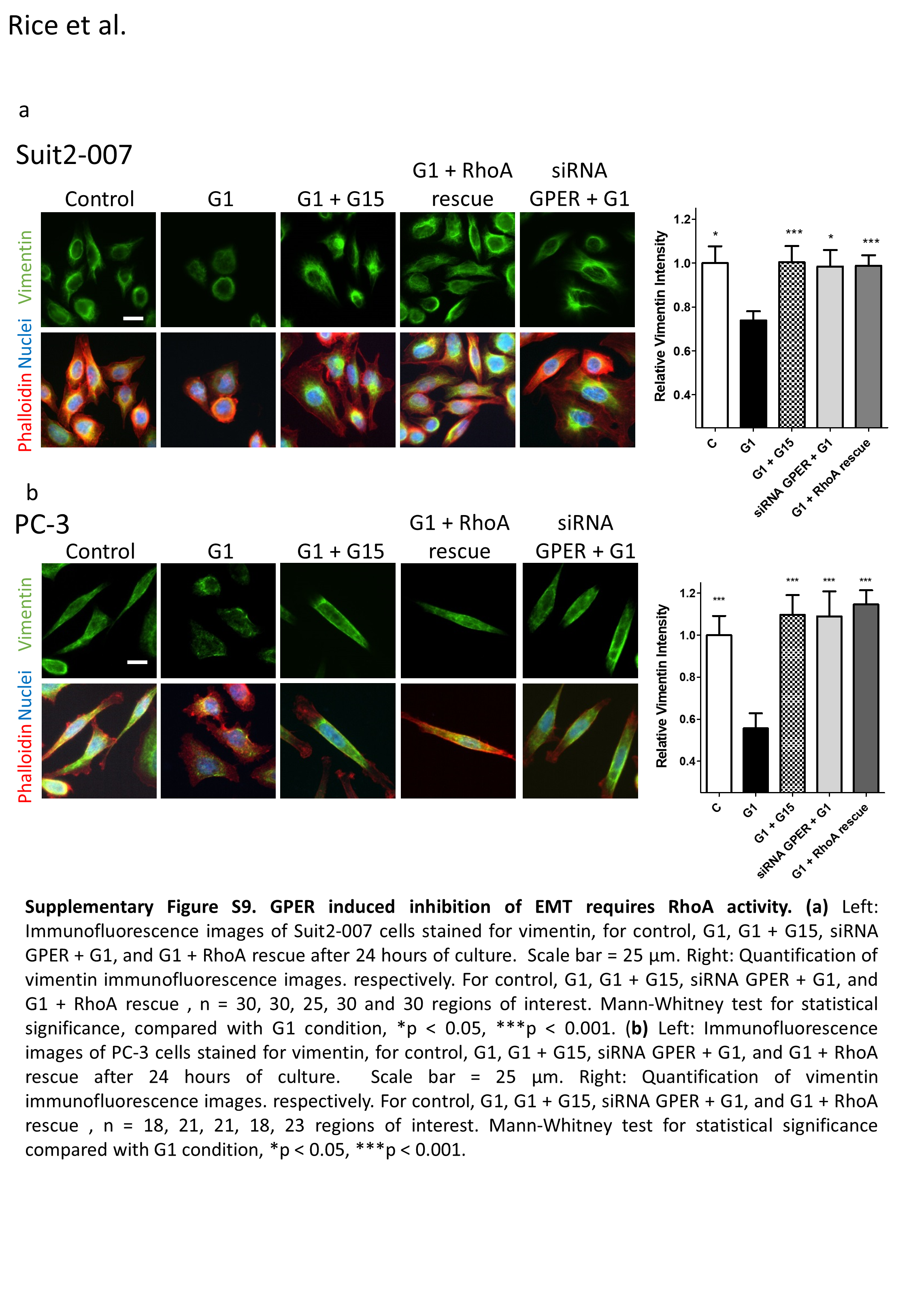

Supplement: Supplementary file 1 [file cancers-12-00289-s001.zip › cancers-679112-supplement/Supplementary Figure S9.TIF]

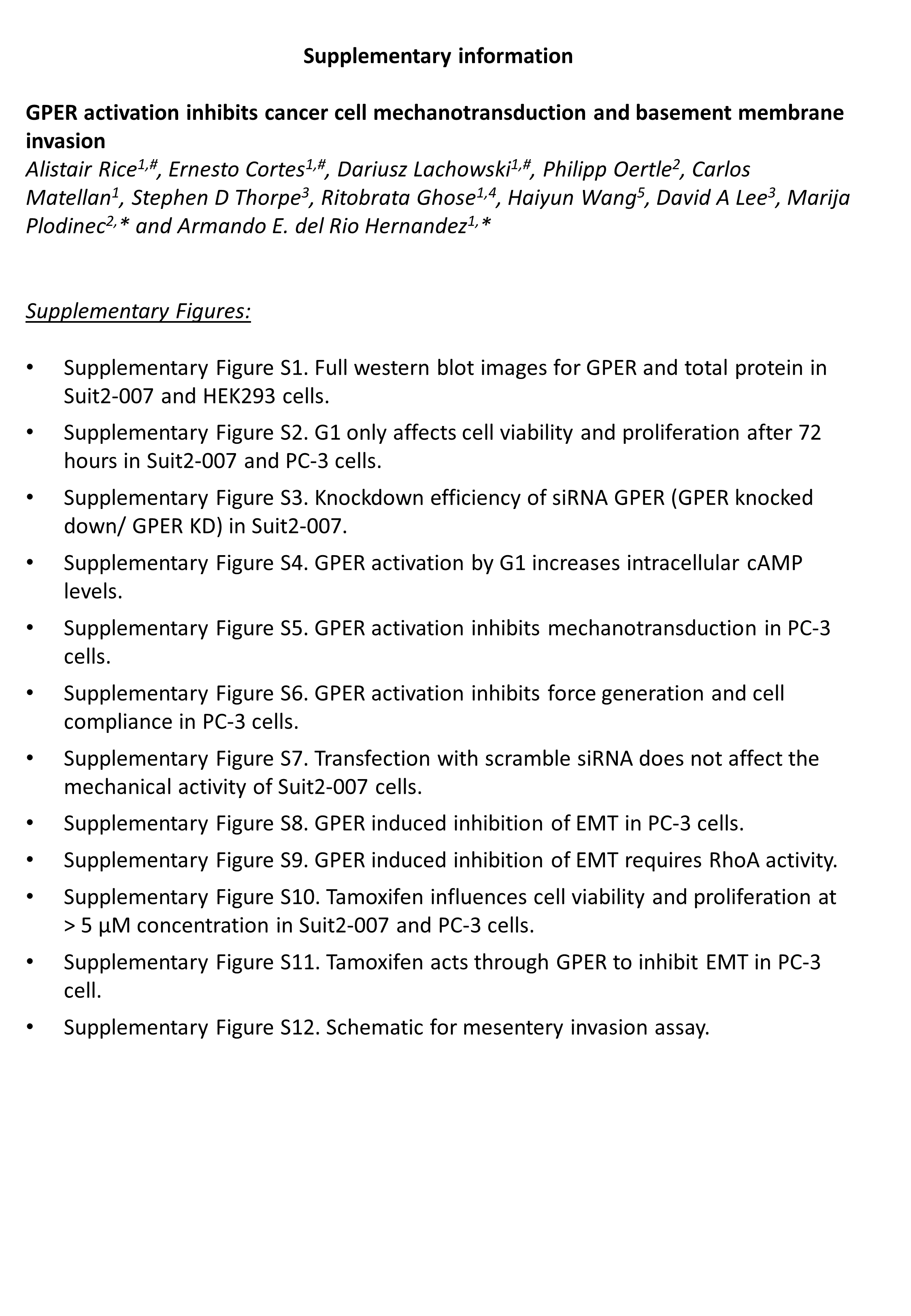

Supplement: Supplementary file 1 [file cancers-12-00289-s001.zip › cancers-679112-supplement/Supplementary Material.TIF]
